# Oral microbiota analyses of paediatric Saudi population reveals signatures of dental caries

**DOI:** 10.1186/s12903-023-03448-3

**Published:** 2023-11-27

**Authors:** Yousef M. Alyousef, Stanley Piotrowski, Faisal A. Alonaizan, Ahmed Alsulaiman, Ali A. Alali, Naif N. Almasood, Chittibabu Vatte, Lauren Hamilton, Divya Gandla, Hetal Lad, Fred L. Robinson, Cyril Cyrus, Ryan C. Meng, Alexa Dowdell, Brian Piening, Brendan J. Keating, Amein K. Al-Ali

**Affiliations:** 1 Department of Preventive Dental Sciences, College of Dentistry, Imam Abdulrahman bin Faisal University, Dammam, Saudi Arabia; 2 Earle A Chiles Research Institute, Providence Cancer Institute, Portland, OR USA; 3 Department of Clinical Biochemistry, College of Medicine, Imam Abdulrahman bin Faisal University, Cornish Road, Rakah, Dammam, 31441 Saudi Arabia; 4 Department of Surgery, Perelman School of Medicine, University of Pennsylvania, Pennsylvania, PA 19104 USA

**Keywords:** Dental caries, 16S rRNA, microbiota, Paediatric, Saudi

## Abstract

**Background:**

Oral microbiome sequencing has revealed key links between microbiome dysfunction and dental caries. However, these efforts have largely focused on Western populations, with few studies on the Middle Eastern communities. The current study aimed to identify the composition and abundance of the oral microbiota in saliva samples of children with different caries levels using machine learning approaches.

**Methods:**

Oral microbiota composition and abundance were identified in 250 Saudi participants with high dental caries and 150 with low dental caries using 16 S rRNA sequencing on a NextSeq 2000 SP flow cell (Illumina, CA) using 250 bp paired-end reads, and attempted to build a classifier using random forest models to assist in the early detection of caries.

**Results:**

The ADONIS test results indicate that there was no significant association between sex and Bray-Curtis dissimilarity (*p* ~ 0.93), but there was a significant association with dental caries status (*p* ~ 0.001). Using an alpha level of 0.05, five differentially abundant operational taxonomic units (OTUs) were identified between males and females as the main effect along with four differentially abundant OTUs between high and low dental caries. The mean metrics for the optimal hyperparameter combination using the model with only differentially abundant OTUs were: Accuracy (0.701); Matthew’s correlation coefficient (0.0509); AUC (0.517) and F1 score (0.821) while the mean metrics for random forest model using all OTUs were:0.675; 0.054; 0.611 and 0.796 respectively.

**Conclusion:**

The assessment of oral microbiota samples in a representative Saudi Arabian population for high and low metrics of dental caries yields signatures of abundances and diversity.

**Supplementary Information:**

The online version contains supplementary material available at 10.1186/s12903-023-03448-3.

## Background

Microorganisms cohabiting within the body are often collectively termed microbiota, and they contribute collectively to host health through means such as synergizing with the immune system, maintaining homeostasis, and production of essential molecules for nutrition and well-being [1]. The human oral cavity is thought to have the second most complex microbial community in the body, after the digestive tract [2]. The surface of teeth is the only non-shedding surface in the oral cavity and allows a stable environment for growth and development of oral microbiota. Two of these key ecosystems are the supragingival and subgingival microbiota which are made up of highly diverse biofilms, but they have been shown to differ greatly in their composition [3, 4].

Dysbiosis of the oral microbiome from a healthy state has been shown to create a number of disease conditions, including dental caries and periodontal disease. Decay damage to a tooth’s surface, or enamel, occurs when bacteria in the oral cavity make acids that attack the enamel. Decay can lead to cavities or dental caries, which if left untreated, can lead to infection and tooth loss. Dental caries is thought to be impacted by supragingival microbiome dysbiosis, while subgingival microbiome dysbiosis is thought to lead to periodontal disease [5, 6]. Handsley-Davis and colleagues recently reported dysbiosis in other oral microbiotas, including saliva, to be linked to dental caries and cause periodontal disease and serious gum infections which may damage the soft tissue and may destroy the bone that supports your teeth [7].

Dental caries is a multifactorial disease process mediated by microbial biofilm and sucrose that causes an unbalance in demineralization and remineralization oral dynamics. Progressive dysbiosis is thought to subsequently cause demineralization of hard dental tissues [8]. Dental caries is highly influenced by diet, mostly through sucrose intake which can impact the ecology of this community by biasing acidogenic and by acid tolerant species of bacteria that are thought to responsible for disease development and progression [9]. Previous research by Høiby et al. indicates that the primary pathogen for dental caries in both the paediatric and adult setting is *Streptococcus mutans* [10]. Second generation sequencing of 16 S ribosomal RNA has identified additional bacteria including *Streptococcus mitis*, *Streptococcus sanguinis* and *Streptococcus oralis*, *Lactobacilli, Actinomyces, Bifidobacteria*, as well as yeast species pathogen with dental caries [5, 11].

Given the scarcity of microbiome studies and the rapidly rising incidence of dental caries in the Saudi population, a large-scale microbiome profiling study of Saudi children with low and high dental caries was conducted. The current study aimed to identify the composition and abundance of the oral microbiota in saliva samples of children with different caries levels using machine learning approaches. This study may assist in the early identification of children who are at higher risk of developing dental caries.

## Methods

### Study subjects

From years 2019–2020, subject data and oral microbiota samples were collected from 400 participants from the Al-Ahssa region of the Eastern Province of Saudi Arabia. Ethical approval of the study was obtained from the Imam Abdulrahman Bin Faisal University Institutional Review Board (IRB) committee (Reference IRB-2019-01-113), and the study was conducted according to the ethical principles of the Declaration of Helsinki and Good Clinical Practice guidelines. Informed written consent in English, with a verified translation in Arabic, was obtained from all participant’s guardians in accordance with the IRB rules. Participants ranged in age from 6 to 12 years old. The children were given a GeneFiXTM Saliva DNA Microbiome Collection tube (MFX-01-Isohelix, UK) which held a stabilizing solution. The guardians of the children were requested to get the children included in the study to spit into the funnel of the tube up to the blue line mark (1ml of unstimulated saliva) prior to ingesting any food or liquid and prior to cleaning their teeth with toothpaste or mouthwash (https://bocascientific.com/images/pdf/genefix-saliva-collectors-brochure-2021.pdf). The guardians were also instructed to shake the tube repeatedly to mix the solution with the sample and then to store the tube at room temperature until collection by a member of the research group.

Examination of all patients by qualified dentists determined the extent of dental caries for each subject in both primary and permanent teeth of each child. The prevalence of dental caries was determined by the Decayed, Missing, and Filled Teeth (DMFT) index, according to the criteria defined elsewhere [12, 13]. The patients were classified according to DMFT score: 400 oral samples from children with dental caries from two categories based on the severity of dental caries were collected [low (DMFT < 4), high (DMFT ≥ 5) [14]. Children with clinically documented dental caries were deemed to satisfy the inclusion criteria. The exclusion criteria for participants were as follows: subjects in an investigational drug evaluation, antibiotic treatment within the previous 30 days and advanced organic disease or haematological disease such as sickle cell anemia.

### DNA library preparation and 16 S rRNA microbiome sequencing

The GeneFiX™ Saliva DNA Microbiome Collection kit (MFX-01-Isohelix, UK) was used for the collection of saliva while bacterial DNA was extracted using Genefix Saliva DNA Isolation kit (Isohelix, UK). We first quantified nucleic acid content in all 400 samples using nanodrop (Thermo Scientific, CA, USA), with all samples having sufficient yield to be brought forward for sequencing. For library preparations, 20 ng of input DNA was used for the assays using the 16 S rRNA Primer Panels. 16 S rRNA gene sequencing is one of the most accurate and widely used methods to identify bacterial phylogeny and genus/species classification.

Libraries were prepared using the Swift Amplicon 16 S rRNA Panel according to the manufacturer’s instructions and including SNAP Combinatorial Dual Indexes for multiplexing (Integrated DNA Technologies [IDT], Coralville, IA, USA). Bead-based library normalization and pooling was performed using Swift Normalase (IDT, Coralville, IA, USA), and representative sets of libraries were assessed for quantity and quality using Qubit fluorometer (Thermo Scientific, CA, USA) and Fragment Analyzer (Agilent Technologies, CA, USA), respectively. A sequencing strategy was employed that targets all variable regions in the 16 S rRNA gene. This was carried out using the Swift Amplicon 16 S rRNA Panel (IDT, Coralville, IA, USA) to enable strain-specific identification of microbial species. The assay utilizes a pool of five overlapping primer pairs for a total targeted area spanning V1-V9, and the resultant libraries are suitable for sequencing on Illumina second generation sequencing platforms. An additional advantage is the large number of sequencing index barcodes available from the Swift Amplicon 16 S Panel, enabling us to multiplex all 400 samples onto a single Illumina flow cell to limit potential batch effects.

For quality control a custom approach was used where polymerase chain reaction (PCR) was performed on each sample using the 515 F primer (forward primer) and one of the 100 × 806rcbc primers (reverse primer). Taq PCR Master Mix from Qiagen was used to prepare the PCR master mix. A PCR reaction was performed on each extracted DNA sample, i.e. with each oral microbiota sample having three PCR reactions performed. The PCR product was run on 1% agarose gel. The indexed libraries were on average 620 base pairs (bp) in length, and individual DNA libraries were diluted to 2.5 nM, pooled in equimolar proportion, and sequenced on a NextSeq 2000 SP flow cell (Illumina, CA) using 250 bp paired-end reads.

### Bioinformatics pre-processing, quality control and filtering

Raw sequences were demultiplexed with Illumina’s bcl2fastq2 v2.20 Seqtk [15]. FastQC was then used for further processing to remove samples with low quality scores across the majority of bases [16]. After de-multiplexing the raw sequences and screening via FastQC, the majority of data processing was executed in QIIME2 with custom scripts. Paired-end reads were joined using the VSEARCH function. Chimera amplicon removal and abundance filtering were processed using Deblur [17, 18]. Amplicon sequences were clustered and assembled into Operational Taxonomical Units (OTUs) using closed reference clustering against the Greengenes (database 13_8) using VESEARCH [19]. Taxonomic assignment was performed using a pre-trained Naïve Bayes classifier with Greengenes OTU database. The abundance tables and data obtained from *QIIME2* were combined into a Phyloseq object, normalized for library size variation using DADA2, and further analysed in R with custom scripts [20].

The resulting taxonomy, OTU abundance, and phylogenetic data from the bioinformatics pre-processing steps were combined into a single object in the R statistical computing environment (v 4.1.1) using the *phyloseq* package (v 1.38.0). The total number of reads per sample that assigned to OTUs using the methods described above were plotted using the *ggplot2* package (v 3.3.5) to identify any samples that sequenced poorly and should be removed (https://ggplot2.tidyverse.org.). The number of samples that would be retained was calculated after applying various read-filtering cut-offs, aiming to maximize the total number of samples in the data set with sufficient counts per OTU. Next, a prevalence filter was applied to remove rare OTUs in order to maximize statistical power in downstream analyses. Prevalence is defined as the number of samples in which a given OTU is observed at least once. Various prevalence filter thresholds were simulated and applied to the data to evaluate the number of distinct OTUs that would be retained with each threshold (Supplementary Fig. 1).

### Evaluating alpha and beta diversity

Alpha diversity, or taxonomic richness, was evaluated between sex and dental caries status groups separately using the unfiltered abundance data following the package authors’ recommendations using the observed richness (i.e., the number of OTUs identified) and two diversity estimators: Shannon entropy and Chao1. The Shannon entropy index takes into account both the number of OTUs present and their proportional abundance. The diversity estimate is maximized when all OTUs have equal relative proportions. As the composition of the community becomes dominated by a single OTU, the estimate approaches zero. When there is only a single OTU in the sample, the estimate is 0. The Chao1 estimator is similar, but places more emphasis on rare taxa. For each estimator, both the raw data and performed rarefaction were examined using the *phyloseq* package and using the sample with the smallest number of reads as the minimum sample size. Quantile-quantile plots for sample quantiles relative to theoretical quantiles from a normal distribution for each data set using the raw data (“none”) and after performing rarefaction (“rarefied”) are shown in Supplemental Fig. 1. Violin plots were used to visualize the distributions of alpha diversity using different estimators and the unfiltered and rarefied data sets, stratified by sex and dental caries status. Additionally, for each data set (raw and rarefied) and estimator (observed richness, Shannon entropy and Chao1 diversity), Welch’s t-tests were employed to evaluate statistical differences in alpha diversity between males and females and high and low dental caries status samples using the *stats* package (v 4.1.1). The false discovery rate procedure (FDR) of Benjamini and Hochberg was used to correct for multiple tests.

The beta diversity and the similarity of communities, were assessed between samples incorporating both taxonomic richness and abundance, using several approaches. First, the Bray-Curtis dissimilarity with the relative abundance of each OTU was calculated using analysis of variance using distance matrices (ADONIS) approach implemented in the *vegan* package (v 2.5.7) to test for statistically significant associations between microbial communities and males and females and high and low dental caries status. This method assumes that if groups are similar in their community compositions, the sums of squares between groups will be greater than the sums of squares within groups. The statistical significance was evaluated using 10,000 permutations. Next, a series of ordinations was performed to visualize meaningful patterns of variation between beta diversity and sex and dental caries status. A principal coordinates analysis (PCoA) was used to visualize the first five principal coordinates using the *ggforce* package (v 0.3.3), with points annotated by both sex and dental caries status.

### Testing for differentially abundant OTUs

Abundance data was agglomerated to the genus level and the package *DESeq2* (v 1.32.0) was employed to identify differentially abundant microbes using sex and dental caries status as covariates in the model. For each comparison (i.e., sex = male vs. female; dental caries status = high vs. low), the *apeglm* log2 fold change shrinkage procedure was used to dampen the effect sizes of OTUs poorly supported by the model. Two different alpha thresholds were used to consider results statistically significant: 0.05 and the 0.1 (the default setting in *DESeq2*). For each comparison and alpha level, volcano plots were created to visualize the effect size of differentially abundant OTUs and heatmaps were created to visualize normalized counts for differentially abundant OTUs stratified by sample metadata.

### Predicting dental caries status with machine learning

An attempt to build a classifier that could accurately predict dental caries status (i.e., high or low) was performed using only the abundance data from the differentially abundant OTUs identified using *DESeq2* (see above). The raw abundance data agglomerated to the genus level were transformed with centred log-ratio approach using the *microbiome* package (v 1.14.0). The *tidymodels* package (v 0.1.4) was used for the machine learning approach (www.tidymodels.org). The data set was split into 70% training and 30% testing sets, stratified by dental caries status. The training set was split into 10-fold cross-validation sets. The *tune* package (v 0.1.6) (https://cran.r-project.org/web/packages/tune/tune.pdf) was used to identify optimal values for two hyperparameters in a random forest model using the *ranger* package (v 0.13.1) (https://cran.r-project.org/web/packages/ranger/citation.html) with 5,000 trees: *mtry*, the number of features to potentially split at each node in an individual the decision tree; and *min_n*, the minimum number of samples per node to control decision tree depth. Twenty different hyperparameter combinations were evaluated using grid search where each parameter combination was used to train and evaluate the fit of a random forest model using 10-fold cross-validation.

As accuracy can be misleading in data sets with unbalanced class sizes, the model fitting during the hyperparameter tuning step was evaluated using the mean area under the ROC curve (AUC) across the 10-fold cross-validation sets for each parameter combination. The hyperparameter set that maximized AUC was used to train a final random forest classifier on the full training set and evaluate model fit using mean AUC, F1 score, and Matthew’s correlation coefficient across the 10-fold cross-validation sets.

To compare the model performance with a limited number of features (i.e., a relatively small number of differentially abundant OTUs) versus using all OTUs as predictors of dental caries status, the same approach described above was used on the full centred log-ratio transformed abundance data, agglomerated to the genus level. A ROC curve was constructed to evaluate the performance of both models over a range of probability thresholds. Finally, a variable importance plot was constructed using the model fitted with all OTUs as predictors to evaluate concordance between the top features selected by the random forest model and those identified using *DESeq2*.

## Results

A histogram showing distribution of reads per sample assigned to OTUs is shown in Supplementary Fig. 3, and scatterplots showing the proportion of samples where a given OTU is observed at least once is illustrated in Supplementary Fig. 4. Supplementary Fig. 5 illustrates the rQuantile-quantile plots rarefaction curves, using the *ggplot2* and *gghighlight* packages, where the number of observed OTUs are plotted as a function of sequencing depth, and shows most individuals have been adequately sampled. Barplots showing taxonomic level and numbers of unclassified or uncultured OTUs are shown in Supplementary Fig. 6. For biological sex and dental caries status, scatterplots showing all combinations of the top five principal components (PCs) identified using broken-stick test methods are shown in Supplementary Figs. 7 and 8, respectively.

### Evaluating alpha and beta diversity

**Fig. 1:**
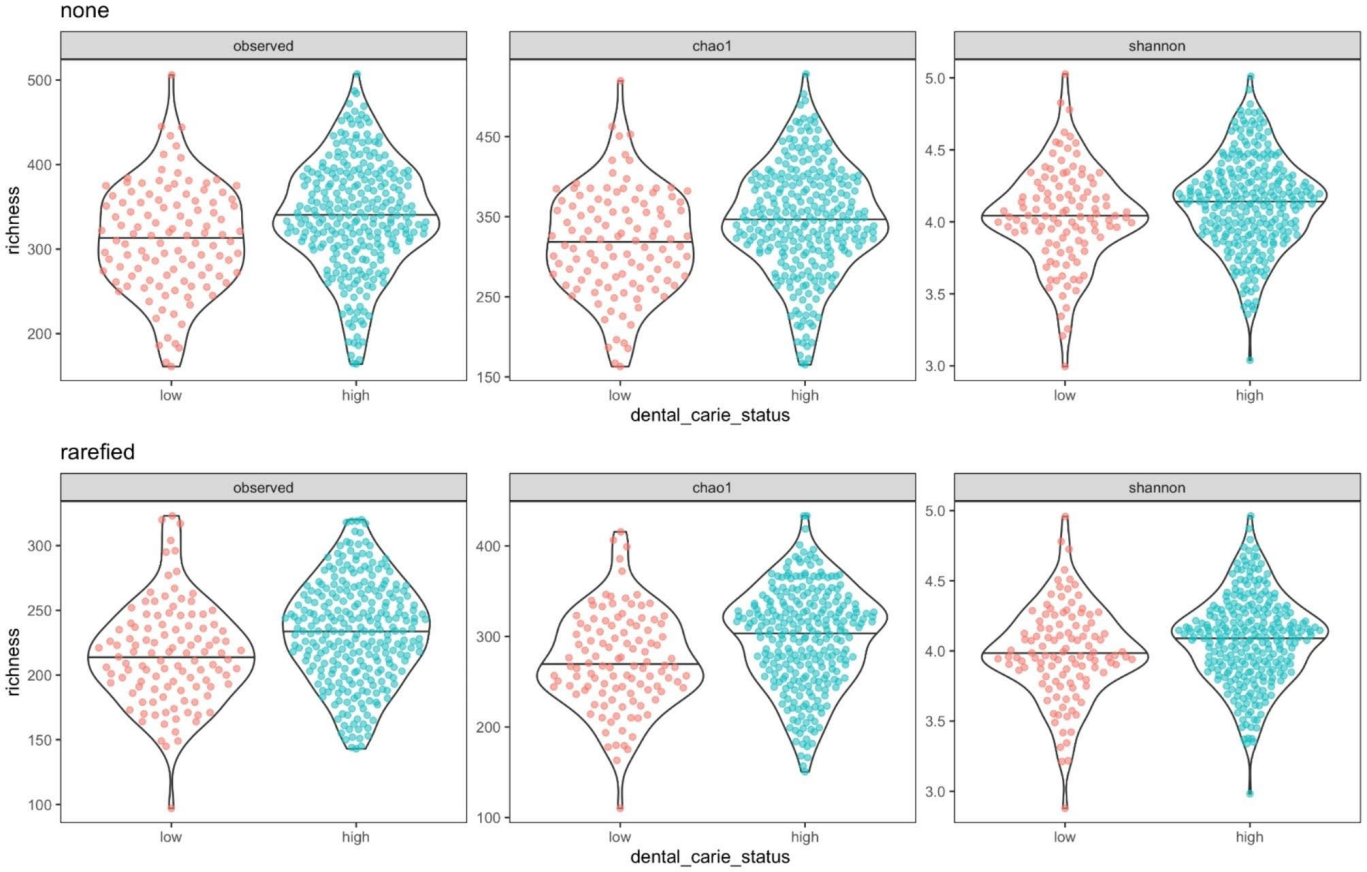
Violin plots showing alpha diversity distribution metrics for low (red) & high (blue) dental caries samples. The median alpha diversity (black horizontal line) and each point (alpha diversity estimate) for a single sample is shown

After correcting for multiple testing, there was no statistical difference in alpha diversity between males and females across data sets (raw and rarefied) and estimators (observed, Shannon entropy, and Chao1 diversity) (Supplementary Figs. 9 and 10). After correcting for multiple testing, high versus low dental caries groups had statistically significant differences in alpha diversity across data sets (observed and rarefied) and estimators (observed, Shannon, and Chao1 diversity) at the 0.05 level. These results are summarized in Table 1 and illustrated in Fig. 1 and Supplementary Fig. 11. Beta-diversity using the Bray-Curtis dissimilarity is illustrated using a heatmap in Supplementary Fig. 11. The ADONIS test results indicate that there was no significant association between sex and Bray-Curtis dissimilarity (*p* ~ 0.93), but there was a significant association with dental caries status (*p* ~ 0.001). However, while the association between the dental caries groups and Bray-Curtis dissimilarity was statistically significant, the effect size was small. The R statistic from the ADONIS test ranges from − 1 to 1, with 0 indicating the groupings are random. The observed statistic from the dental caries group is 0.08041.

**Table 1:** Welch’s t-tests comparing alpha diversity metrics across Saudi Caries datasets. Variable column defines groups: sex: male or female; Caries: low or high; transformation, none (unfiltered), rarefied (rarefaction to even depth)

Variable	Transformation	Estimator	Estimate	Statistic	Conf low	Conf high	P value	P-adjusted
Sex	none	Observed	2.915	0.364	-12.878	18.708	0.716	0.818
Sex	none	Chao1	3.531	0.429	-12.74	19.801	0.669	0.818
Sex	none	Shannon	0.002	0.042	-0.072	0.075	0.966	0.966
Sex	none	Simpson	-0.002	-0.784	-0.007	0.003	0.434	0.772
Sex	rarefied	Observed	2.063	0.426	-7.506	11.633	0.671	0.818
Sex	rarefied	Chao1	4.032	0.6	-9.239	17.302	0.549	0.799
Sex	rarefied	Shannon	0.005	0.143	-0.067	0.078	0.887	0.946
Sex	rarefied	Simpson	-0.002	-0.67	-0.006	0.003	0.504	0.799
Caries status	none	Observed	-26.67	-3.741	-40.723	-12.618	0	0.001
Caries status	none	Chao1	-27.307	-3.703	-41.84	-12.773	0	0.001
Caries status	none	Shannon	-0.096	-2.608	-0.169	-0.023	0.01	0.028
Caries status	none	Simpson	-0.003	-0.983	-0.008	0.003	0.327	0.654
Caries status	rarefied	Observed	-16.754	-3.85	-25.331	-8.176	0	0.001
Caries status	rarefied	Chao1	-25.386	-4.236	-37.199	-13.573	0	0.001
Caries status	rarefied	Shannon	-0.094	-2.588	-0.166	-0.022	0.01	0.028
Caries status	rarefied	Simpson	-0.003	-1.109	-0.009	0.002	0.269	0.615

### Differential abundance testing

**Fig. 2:**
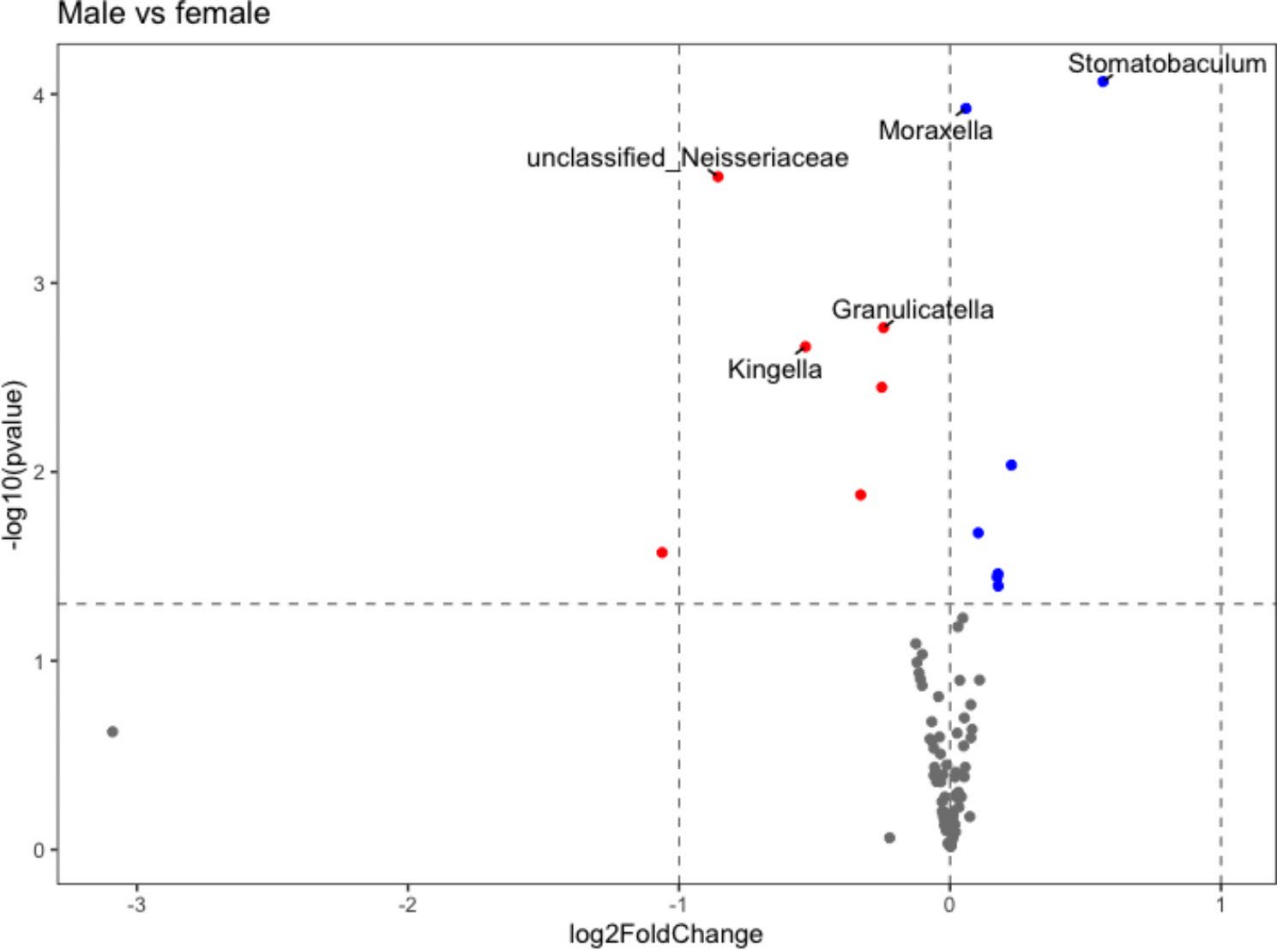
Volcano plot illustrating the change estimates of each OTU with sex as the main effect (p-value alpha = 0.05). Log2 fold change is presented on x-axis; with -log10 transformed raw p-value presented on y-axis. Grey dots are not statistically significant; red dots are statistically significant (raw p-value alpha level of 0.05) with negative log2 fold changes; blue dots are statistically significant at raw p-value level of 0.05 with positive log2 fold changes. Labelled dots are statistically significant (adjusted p-value alpha of 0.05)

**Fig. 3:**
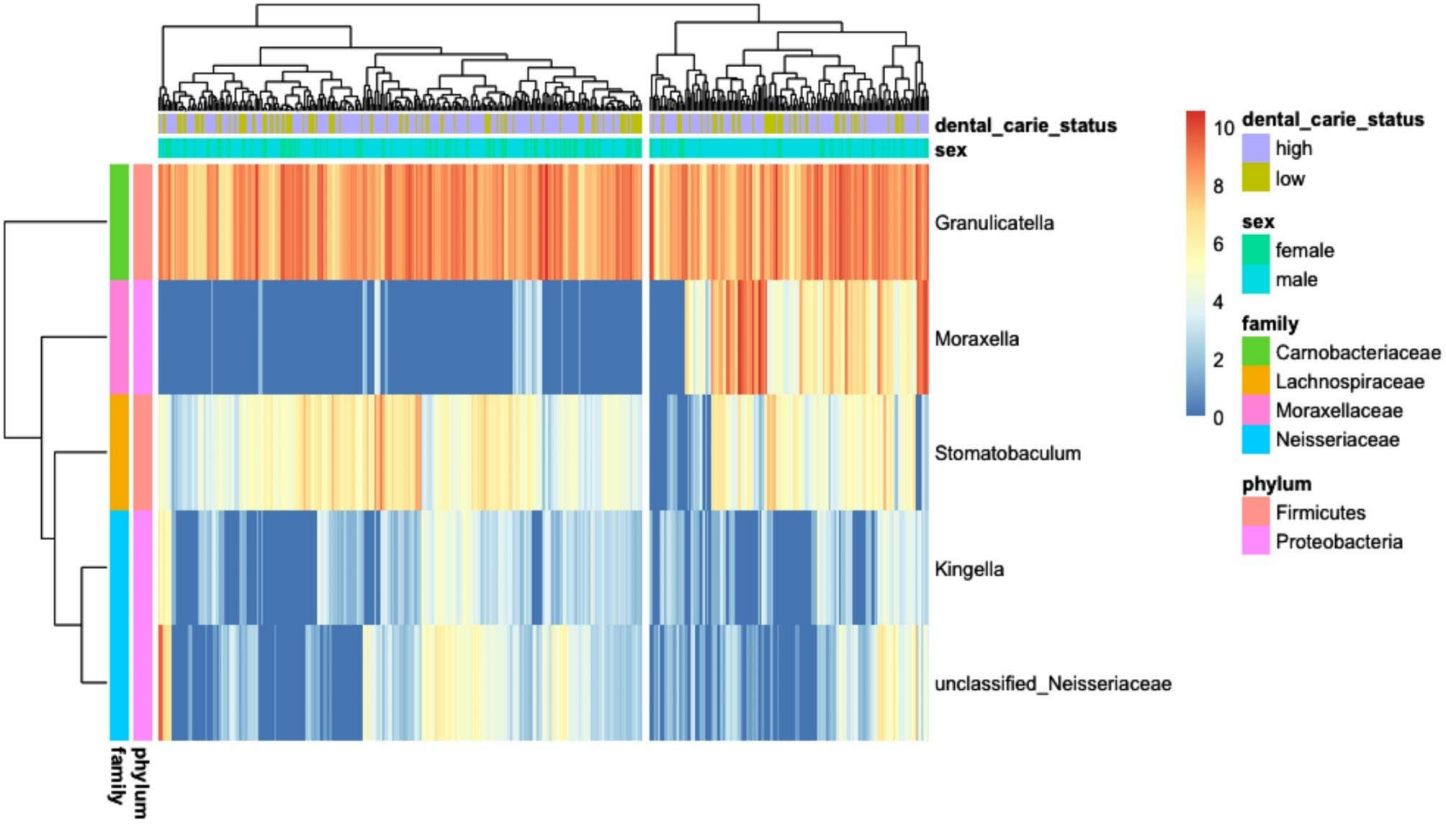
Heatmap of differentially abundant OTUs identified using DESeq2 in the Saudi dental caries population. (adjusted p-value alpha of 0.05). Samples are clustered on top dendrogram, with OTUs clustered on the left dendrogram

Bar plots showing the relative abundance of each phylum for males and females are shown in Supplementary Fig. 12. Using an alpha level of 0.05, five differentially abundant OTUs were identified between males and females as the main effect (Table 2), and Figs. 2 and 3 summarize and illustrate these results using violin plots and heatmaps, respectively. Supplementary Table 1 shows the equivalent datasets using an alpha level of 0.1 (the default setting in DESeq2). Six differentially abundant OTUs between males and females as the main effect were identified along with four differentially abundant OTUs between high and low dental caries.

**Table 2:** Differentially abundant OTUs identified using DESeq2 at the alpha = 0.05 level between males and females as the main effect and differentially abundant OTUs identified using DESeq2 in high vs. low dental caries status (alpha = 0.1 level). The taxonomic information of each identified OTU, estimated log2 fold change (LFC), and adjusted p-value using the FDR approach (p value adjusted) are reported

Status	Phylum	Class	Order	family	Genus	LFC	P value adjusted
Males versus females	Proteobacteria	Gammaproteobacteria	Burkholderiales	Neisseriaceae	Kingella	-0.533	0.037
	Firmicutes	Clostridia	Lachnospirales	Lachnospiraceae	Stomatobaculum	0.565	0.005
	Firmicutes	Bacilli	Lactobacillales	Carnobacteriaceae	Granulicatella	-0.245	0.037
	Proteobacteria	Gammaproteobacteria	Burkholderiales	Neisseriaceae	Unclassified Neisseriaceae	-0.856	0.008
	Proteobacteria	Gammaproteobacteria	Pseudomonadales	Moraxellaceae	Moraxella	0.059	0.005
High versus low dental caries	Firmicutes	Negativicutes	Veillonellales-Selenomonadales	Selenomonadaceae	Uncultured	-0.001	0.071
	Firmicutes	Negativicutes	Veillonellales-Selenomonadales	Veillonellaceae	Unclassified Veillonellaceae	0.03	0.071
	Firmicutes	Clostridia	Lachnospirales	Lachnospiraceae	Oribacterium	-0.278	0.071
	Firmicutes	Bacilli	Lactobacillales	Unclassified Lactobacillales	Unclassified Lactobacillales	0.409	0.076

**Fig. 4:**
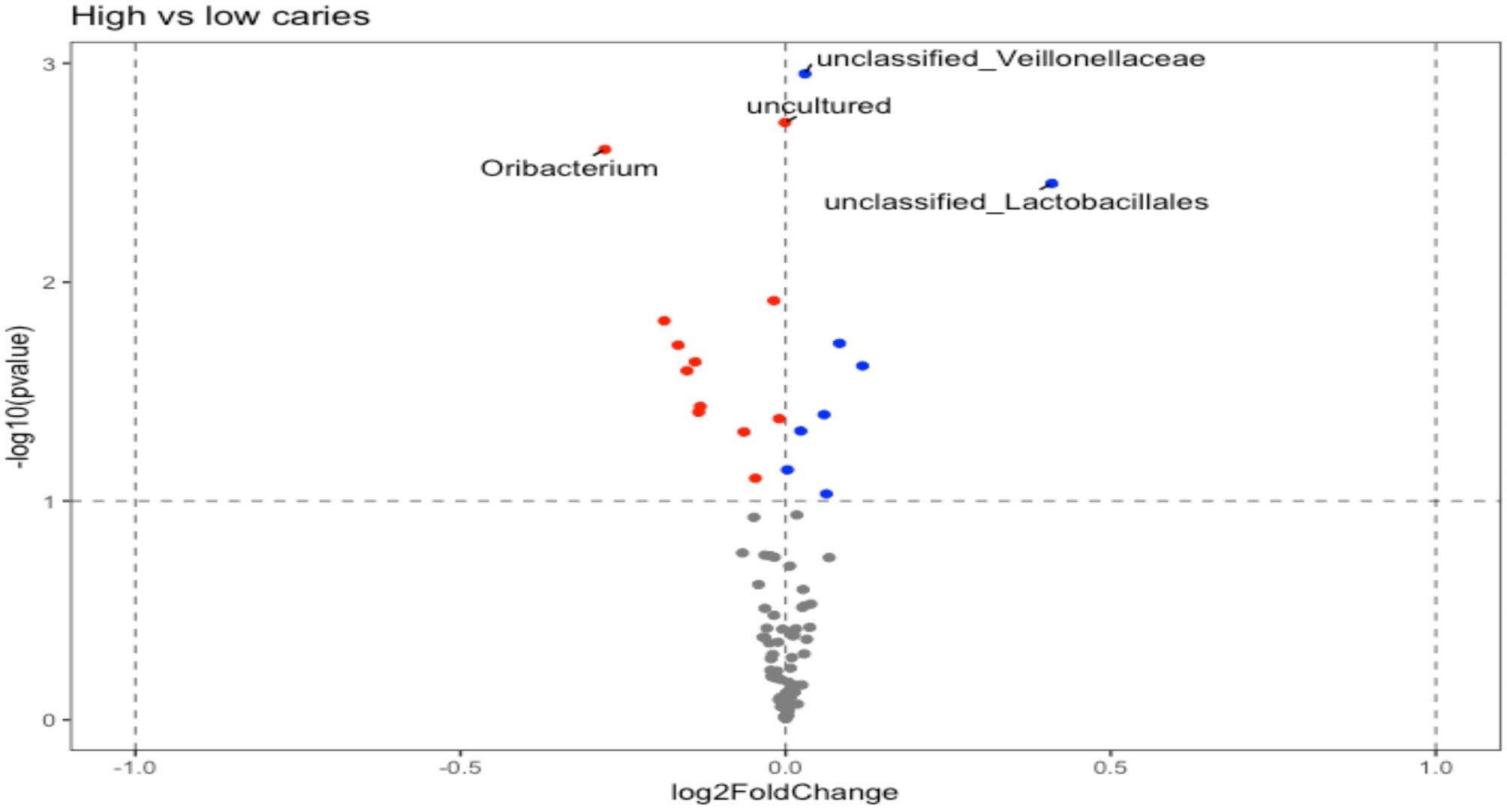
Volcano plot showing log2 fold change estimates of each OTU with High vs. Low Dental Caries status. Log2 fold changes shown on x-axis; log10 transformed raw p-value shown on y-axis. Red dots are statistically significant (raw p-value alpha level of 0.1) with negative log2 fold changes; blue dots are statistically significant (raw p-value level of 0.1) with positive log2 fold changes; grey dots not statistically significant. Labelled points are statistically significant at adjusted p-value alpha of 0.1

**Fig. 5:**
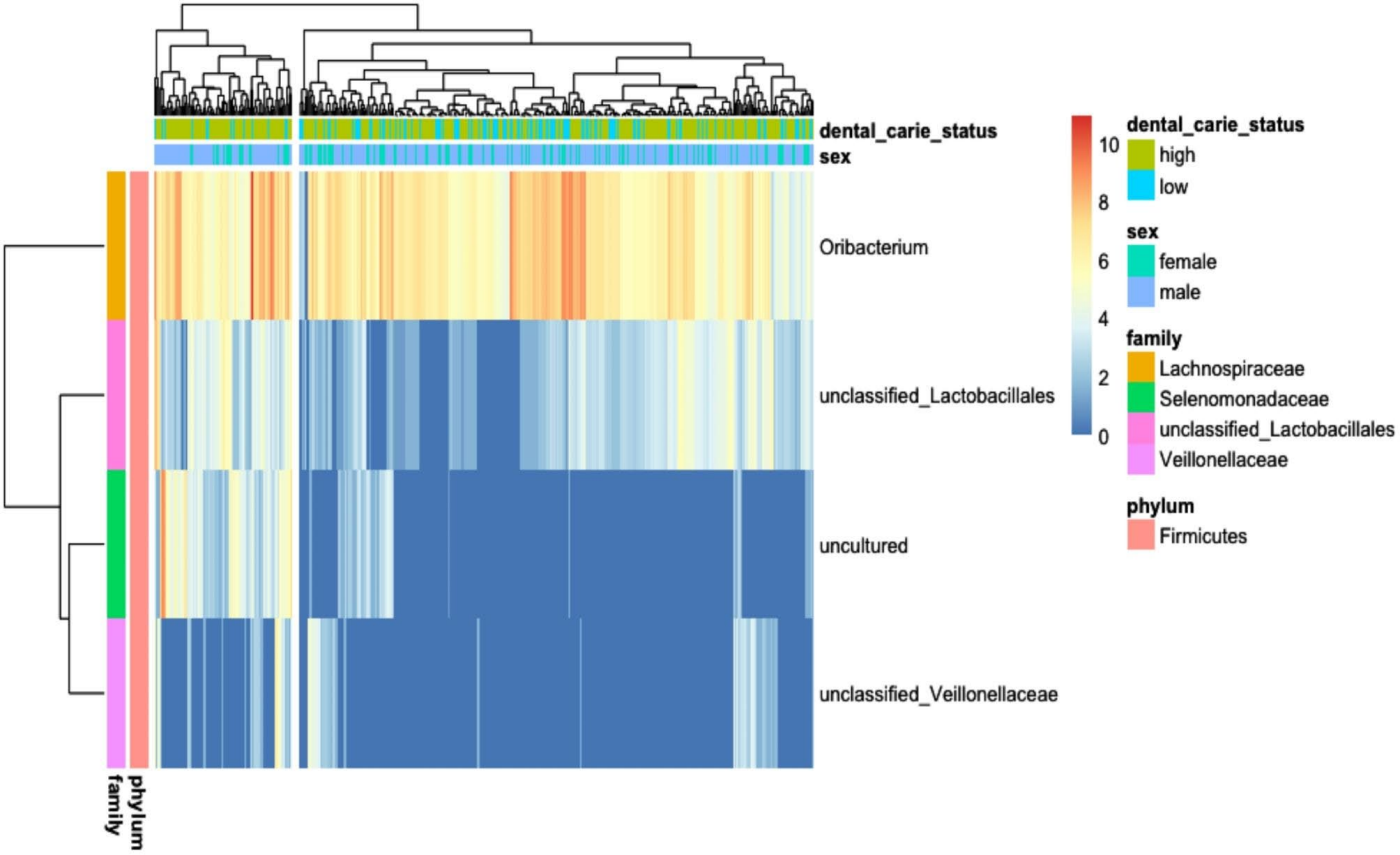
Heatmap of differentially abundant OTUs for dental caries status identified using DESeq2 (adjusted p-value alpha of 0.1). Samples are clustered on the top dendrogram; OTUs are clustered on the left dendrogram

Differentially abundant OTUs between high and low dental caries status are shown in Table 2 using an alpha level of 0.1. Figures 4 and 5 illustrate these results using violin plots and heatmaps, respectively.

### Random Forest Classifiers

**Fig. 6:**
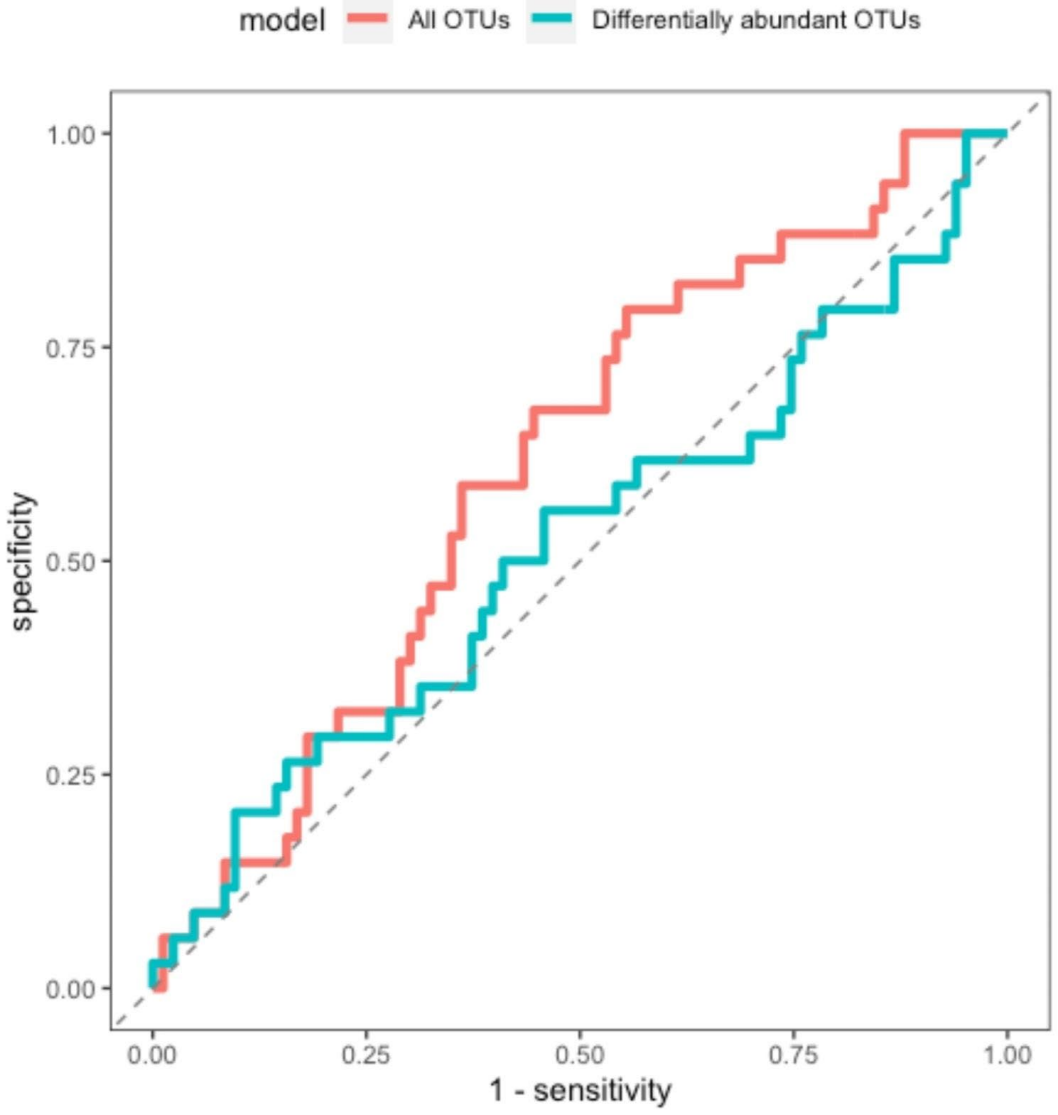
ROC curve. The curve showing the performance of each random forest model across probability thresholds for dental caries status prediction

Scatterplot illustrations of the mean AUC of different hyperparameter combinations in a random forest model using differentially abundant OTUs (Supplementary Fig. 13a) as well as all OTUs (Supplementary Fig. 13b) as features using 5,000 trees was conducted. In Fig. 6, the ROC curve illustrates the performance of each random forest model for dental caries status prediction across the spectrum of probability thresholds. Supplementary Fig. 14 overviews the variable importance plot by illustrating the top 10 features in the random forest model using all OTUs as features to predict dental caries status. The mean metrics for the optimal hyperparameter combination using the model with only differentially abundant OTUs were as follows with estimates in brackets: Accuracy (0.701); Matthew’s correlation coefficient (0.0509); AUC (0.517) and F1 score (0.821). The mean metrics for random forest model using all OTUs with estimates in brackets were as follows: Accuracy (0.675); Matthew’s correlation coefficient (0.054); AUC (0.611); F1 score (0.796).

## Discussion

Al-Shammery and colleagues performed the first national study of dental caries in Saudi Arabia in 1999. In this study, they assessed 12–13 year-old subjects (n = 1873) in 10 of the 13 regions of Saudi Arabia and reported a prevalence of 74% and 67% in urban and rural populations, respectively [21]. In 2010, Al Dosari et al. assessed representative 6–7, 12–13 and 15–18 year-old children across 11 regions of Saudi Arabia in a larger study (n = 12,200) and observed a dental caries prevalence of 59–80%, depending on the fluoride levels in the drinking water within each given region [22]. High carbohydrate consumption combined with poor oral hygiene is considered to be the main cause of severe dental caries. The advent the 16s rRNA sequencing of salivary microbiota has made it possible to provide in-depth information on the composition of oral microbiota, including rare and uncultivated species, which aids in the understanding of the pathological effects of microbiota in relation to the development of dental caries. Thus, the identification of the composition of the oral microbiome has remained a key topic in dental caries research. as the identification of caries-associated microbes has the potential to lead to the identification of children who are at higher risk of developing dental caries by, caries-prevention treatments via the alteration of the oral microbiome. This is especially important in paediatric populations who are still developing skills for dental hygiene and early intervention is especially critical to establish lifelong hygiene practices.

In our study ,there were no statistical differences in alpha diversity observed between males and females across data sets using standard estimates, including standard Shannon entropy and Chao1 diversity approaches, which has been reported in other studies, including a study conducted in the neighbouring country of Qatar [23, 24]. However, the high versus low dental caries groups had statistically significant differences in alpha diversity using the same approaches. Although many studies have reported similar results, others reported no significant difference in alpha diversity between low and high dental caries [25]. This discrepancy may be attributed to small sample size and the subsequent classification of the study participants into numerous groups which resulted in loss of statistical power. To assess Beta diversity, we utilized ADONIS testing which showed no significant associations between sex and Bray-Curtis dissimilarity, but statistically significant associations with dental caries status were evident (p ~ 0.001), which is aligned with the study conducted by Butcher et al. [25]. While the association between the dental caries groups and Bray-Curtis dissimilarity were significant, the effect size was modest.

Differentially abundant microbiota were identified between males and females, which again is in line with other studies [23]. Species resolution was not achievable on the Kingella genus finding, although there are only four recognized species in the Kingella genus [26]. The K. oralis species is found in the oral microbiome and is associated with dental plaque [27]. Closer examination of the taxonomy table for this OTU shows it is currently defined down to the species-level as K. dentrificans. For the Stomatobaculum genus observation, there is only one characterized species, S. longum, in this genus, which has been reported to be present in subgingival dental plaque, although there are no reports to date with dental caries. S. longum is a strict anaerobic Gram-stain-variable that is able to grow on various substrates, including yeast extract, some sugars and organic acids [27]. The 16 S rRNA gene sequence phylogeny of several uncultured microbiota from a separate branch within the Lachnospiraceae family with the strongest sequence homology to Moryella were also identified. Children who consume large quantities of sugar-sweetened drinks have been reported to have lower Moryella genus levels [27]. The Granulicatella genus observation is unclassified at the species level. Granulicatella has been shown to be increased in abundance in Indian and Chinese children with high dental caries [28, 29]. The Neisseriaceae family also includes the Kingella genus. Higher sequencing depth may resolve the finding at least to the genus level, which may indicate K. oralis. The Moraxella observation is also unclassified at the species level, but children drinking more sugar-sweetened drinks have been reported to have lower levels of Moraxella genus levels [30]. The Lactobacillales association has also not been characterized down to the family and genus levels, but part of the order of acid-tolerant microbial taxa associated with tooth decay and periodontal disease has been reported [31].

A number of differentially abundant OTUs were observed to be significant between high and low dental caries status. The Selenomonadaceae family observation is consistent with an over-representation of a severe caries cohort in a 2020 Indian study conducted by Kalpana et al. [32]. In addition a report by Lin et al. identified the Selenomonadaceae family to be a potential biomarker for high dental caries subjects and exhibited a positive correlation with DMFT [24]. Moreover, Tang et al. showed that the anaerobic glycolytic pathway was significantly upregulated in the high dental caries group in unison with upregulation of these bacterial families, which leads to elevated acid production, thereby overwhelming the healthy microbiome capacity for buffering [14, 33, 34]. The observed association of dental caries with the Veillonellaceae family is also consistent with a significant enrichment in a 2017 dental caries study conducted by Eriksson and colleagues on 63 Swedish teenagers [35]. Although it should be noted that similar to the Selemonadaceae observation, the effect size was very low. Again, the hypothesis is that Veillonella are anaerobic gram-negative bacteria which may serve as an ‘acid sink’ which provides the appropriate environment for the growth of acidogenic bacteria, such as the Streptococcus species which will augment acid production [36, 37]. A significant association with the Oribacterium genus was also observed. Abundance of O. parvum has also been associated with a severe dental caries population [32]. Given the relatively low effect size, the utility of microbiome signatures as a clinical biomarker in this population may be somewhat limited, however we note that 16 S sequencing performed here is typically more limited in species-level detection versus shotgun metagenomic sequencing. Given these limitations, the latter approach may have utility in this context.

We also used a random forest machine learning approach to construct a classifier to examine high versus low dental caries by dividing the dataset into training and testing portions. We trained the classifier based on differential microbes from the diversity and abundance components of the study and assessed performance in classifying high versus low dental caries in the held-out testing set. In general, model fitting using differentially abundant OTUs increased as the minimum number of samples to split at each node increased. In general, the classifier did not perform much better than random chance. The mean AUC for the best model fit was 0.573 and the Matthew’s correlation coefficient was 0.274. The relatively poor fit of the model could be due in part to class imbalance, with approximately 70% of the filtered data set composed of high dental caries samples compared to 30% low dental caries samples, although these proportions are not too out of balance when compared to many microbiota studies. Strategies for potentially improving such a classifier include utilizing shotgun metagenomic data, increased and balanced study population sizes and potentially deeper sequencing to identify lower abundance microbial populations.

It was a major limitation that we were unable to collect deeper dental caries-related phenotypes and relevant covariates in this paediatric study population. However, attempts are being made to get assent and consent from these individuals as they progress through the Eastern Province school system. Such prospective longitudinal data may yield insights in age-related microbiota dynamics especially within a society that has experienced profound changes in diet and lifestyle. In addition, the children’s dietary and oral hygiene habits and their behaviour were not taken into consideration. However, as the children were all from the same geographical area of Saudi Arabia, one would assume that they would have similar dietary and oral hygiene habits and similar behaviours.

## Conclusion

In conclusion, assessment of oral microbiota samples in a representative Saudi Arabian population for high and low metrics of dental caries yields signatures of abundances and diversity which may assist in the early identification of children who are at higher risk of developing dental caries.

### Electronic supplementary material

Below is the link to the electronic supplementary material.

Supplementary Material 1

Supplementary Material 2

Supplementary Material 3

Supplementary Material 4

Supplementary Material 5

Supplementary Material 6

Supplementary Material 7

Supplementary Material 8

Supplementary Material 9

Supplementary Material 10

Supplementary Material 11

Supplementary Material 12

Supplementary Material 13

Supplementary Material 14

Supplementary Material 15

## Data Availability

The datasets generated during the current study are available in the European Nucleotide Archive (ENA) repository, https://www.ebi.ac.uk/ena/browser/home, under the title” Oral Microbiota Analyses of Paediatric Saudi Population Reveals Signatures of Dental Caries” with accession number PRJEB57557. All requests for data can be sent to the corresponding author (AKA) and verified academic investigators will be granted full access.

## List of Abbreviations

OTUs: Operational taxonomic units
DMFT: Decayed, Missing, and Filled Teeth
PCR: Polymerase chain reaction
PCoA: Principal coordinates analysis
AUC: Area under the curve
PCs: Principal components
