# Supplementary material for: Oral microbiota analyses of paediatric Saudi population reveals signatures of dental caries
Source: BMC Oral Health. 2023 Nov 27;23:935. doi: 10.1186/s12903-023-03448-3 (PMC10683298; doi:10.1186/s12903-023-03448-3)

Supplementary Figure 1. Quantile-quantile plots for sample quantiles relative to theoretical quantiles from a normal distribution for each data set using the raw data (“none”) and after performing rarefaction (“rarefied”). Dashed diagonal black lines represent perfect concordance between samples quantiles and theoretical quantiles from a normal distribution.

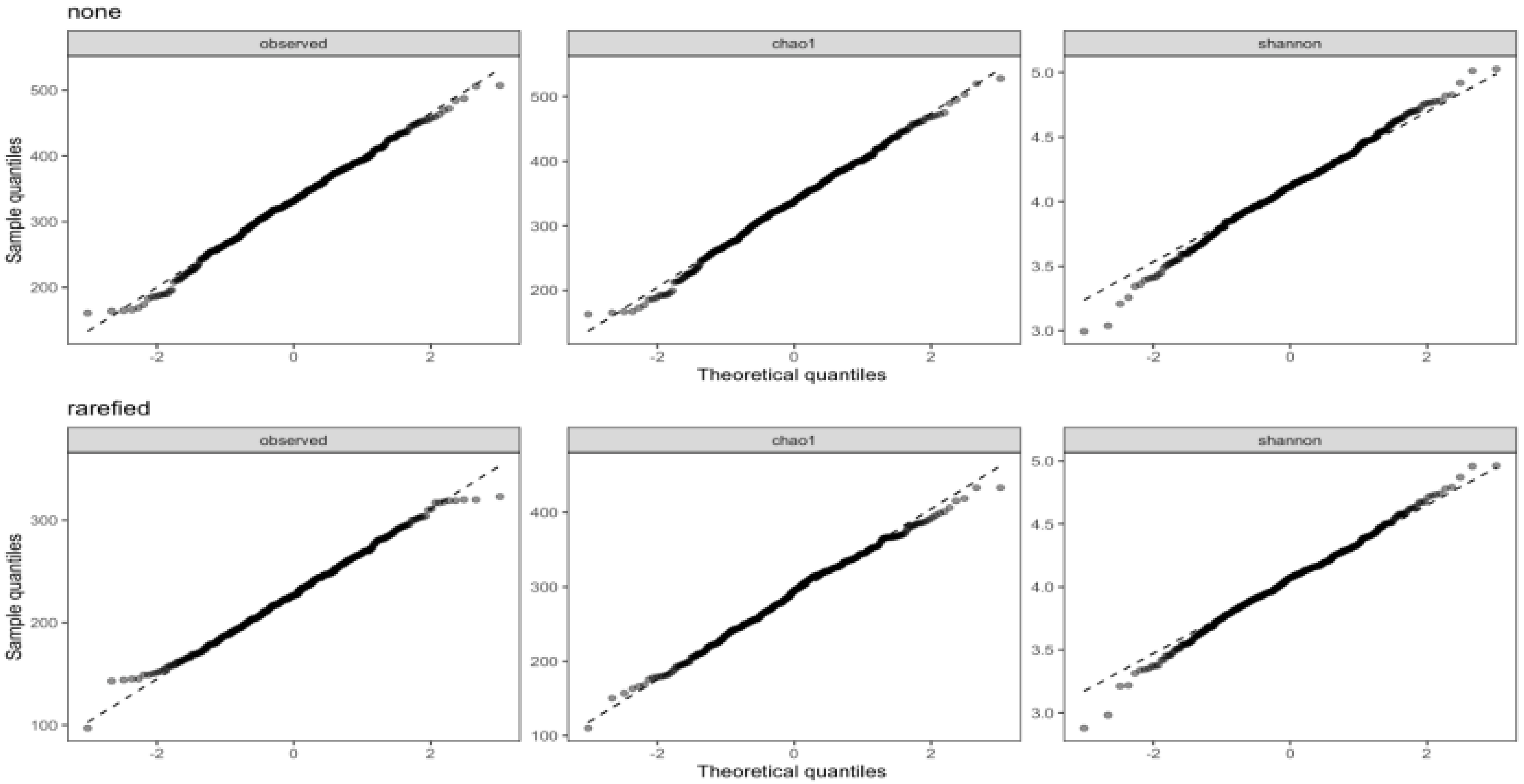

Supplement: Supplementary file 1 — Supplementary Material 1 [file 12903_2023_3448_MOESM1_ESM.pdf]
