# Supplementary material for: Oral microbiota analyses of paediatric Saudi population reveals signatures of dental caries
Source: BMC Oral Health. 2023 Nov 27;23:935. doi: 10.1186/s12903-023-03448-3 (PMC10683298; doi:10.1186/s12903-023-03448-3)

Supplementary Figure 2. Bar plot showing each simulated prevalence filter threshold on x-axis and corresponding number of retained OTUs on y-axis. A prevalence filter of 0 means that all OTUs are retained; a prevalence filter of 1 means that only OTUs observed at least once in every sample are retained.

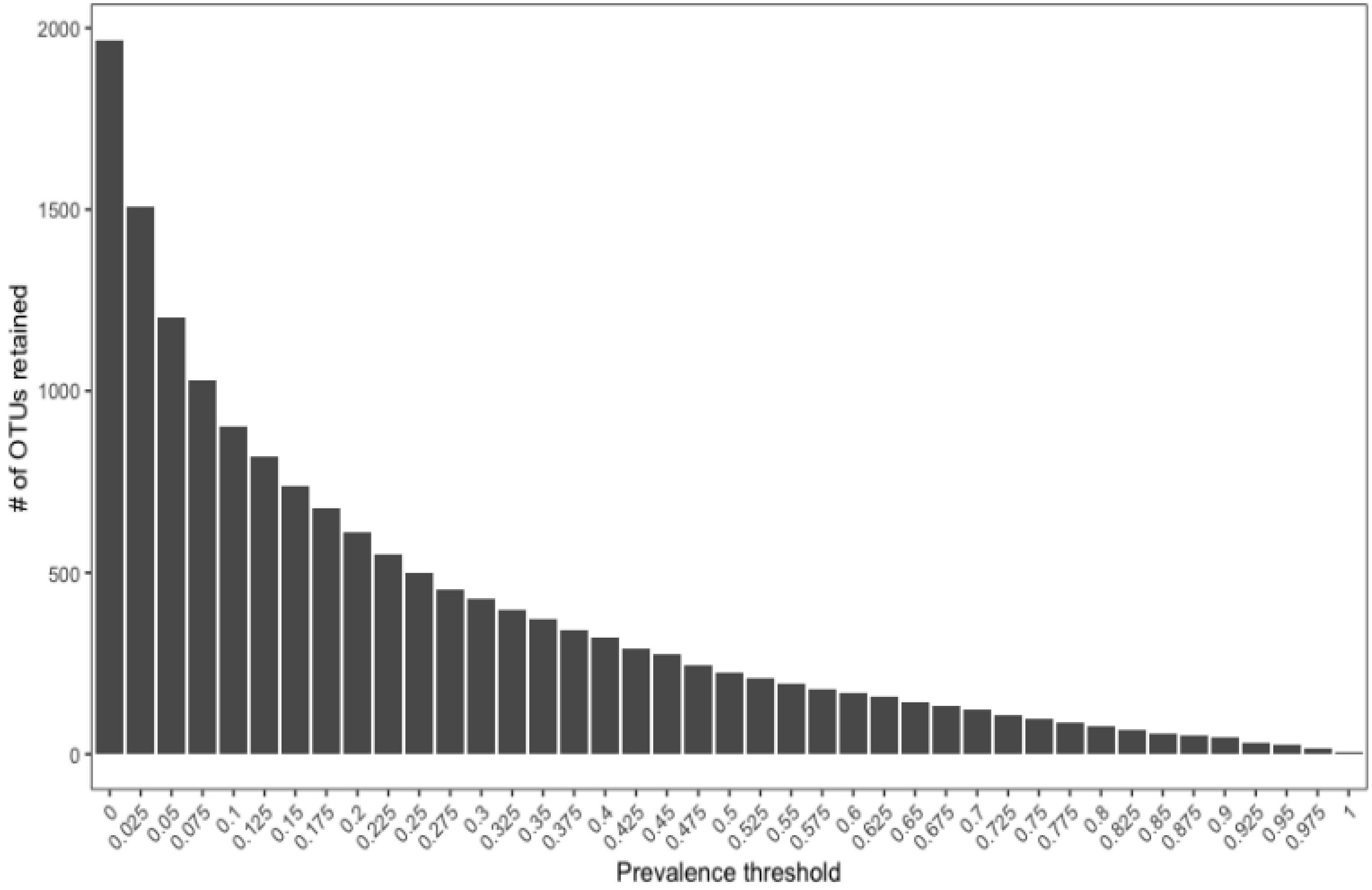

Supplement: Supplementary file 2 — Supplementary Material 2 [file 12903_2023_3448_MOESM2_ESM.pdf]
