# Supplementary material for: Oral microbiota analyses of paediatric Saudi population reveals signatures of dental caries
Source: BMC Oral Health. 2023 Nov 27;23:935. doi: 10.1186/s12903-023-03448-3 (PMC10683298; doi:10.1186/s12903-023-03448-3)

Supplementary Figure 6. Barplots showing taxonomic level (x-axis) and numbers of unclassified or uncultured OTUs (y-axis)

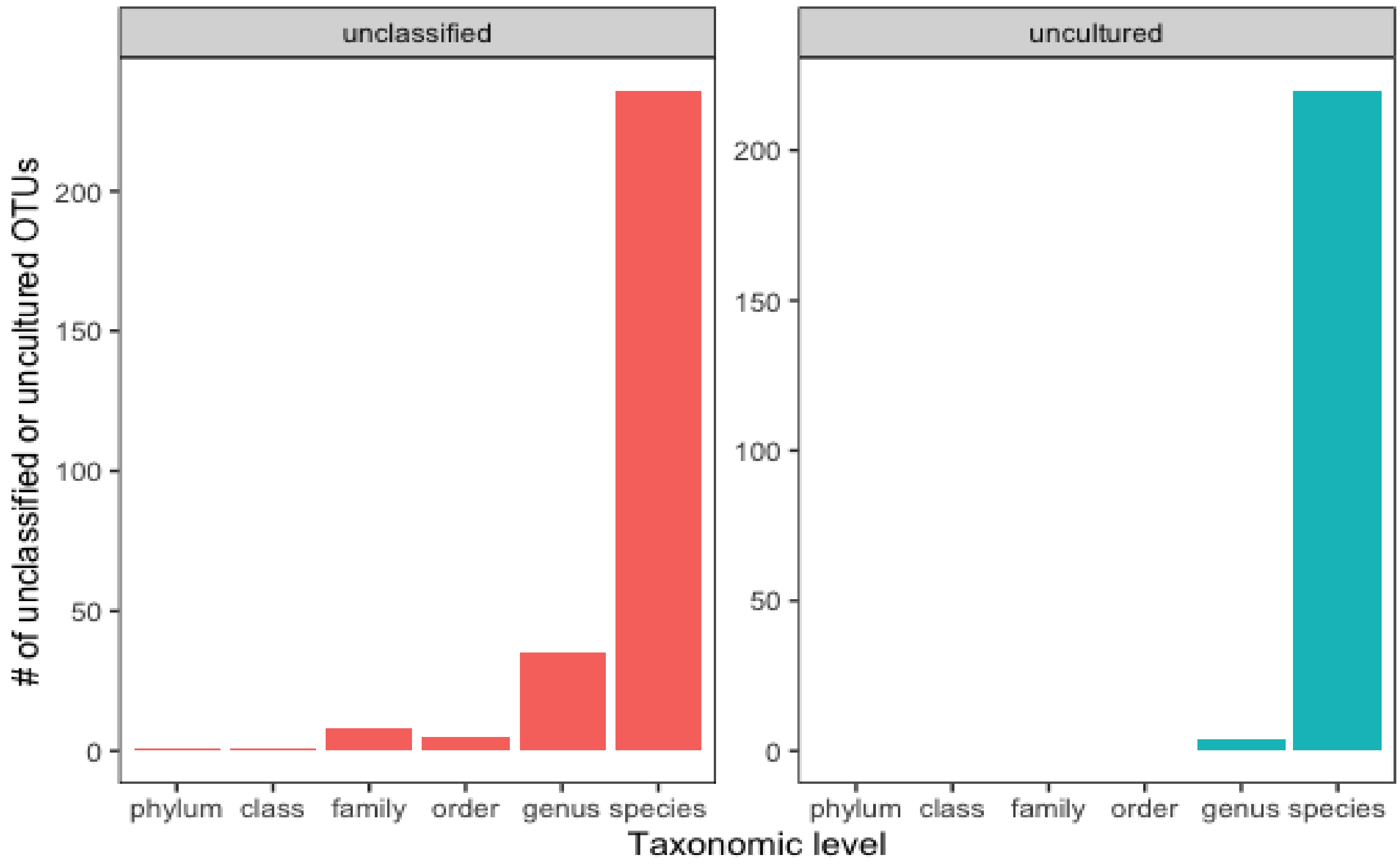

Supplement: Supplementary file 3 — Supplementary Material 3 [file 12903_2023_3448_MOESM3_ESM.pdf]
