# Supplementary material for: Oral microbiota analyses of paediatric Saudi population reveals signatures of dental caries
Source: BMC Oral Health. 2023 Nov 27;23:935. doi: 10.1186/s12903-023-03448-3 (PMC10683298; doi:10.1186/s12903-023-03448-3)

Supplementary Figure 9. Violin plots showing the distribution of alpha diversity metrics between **males (red) and females (blue)** across for each data set and estimator. In each violin plot, the black horizontal line is the median alpha diversity and each point is the alpha diversity estimate for a single sample.

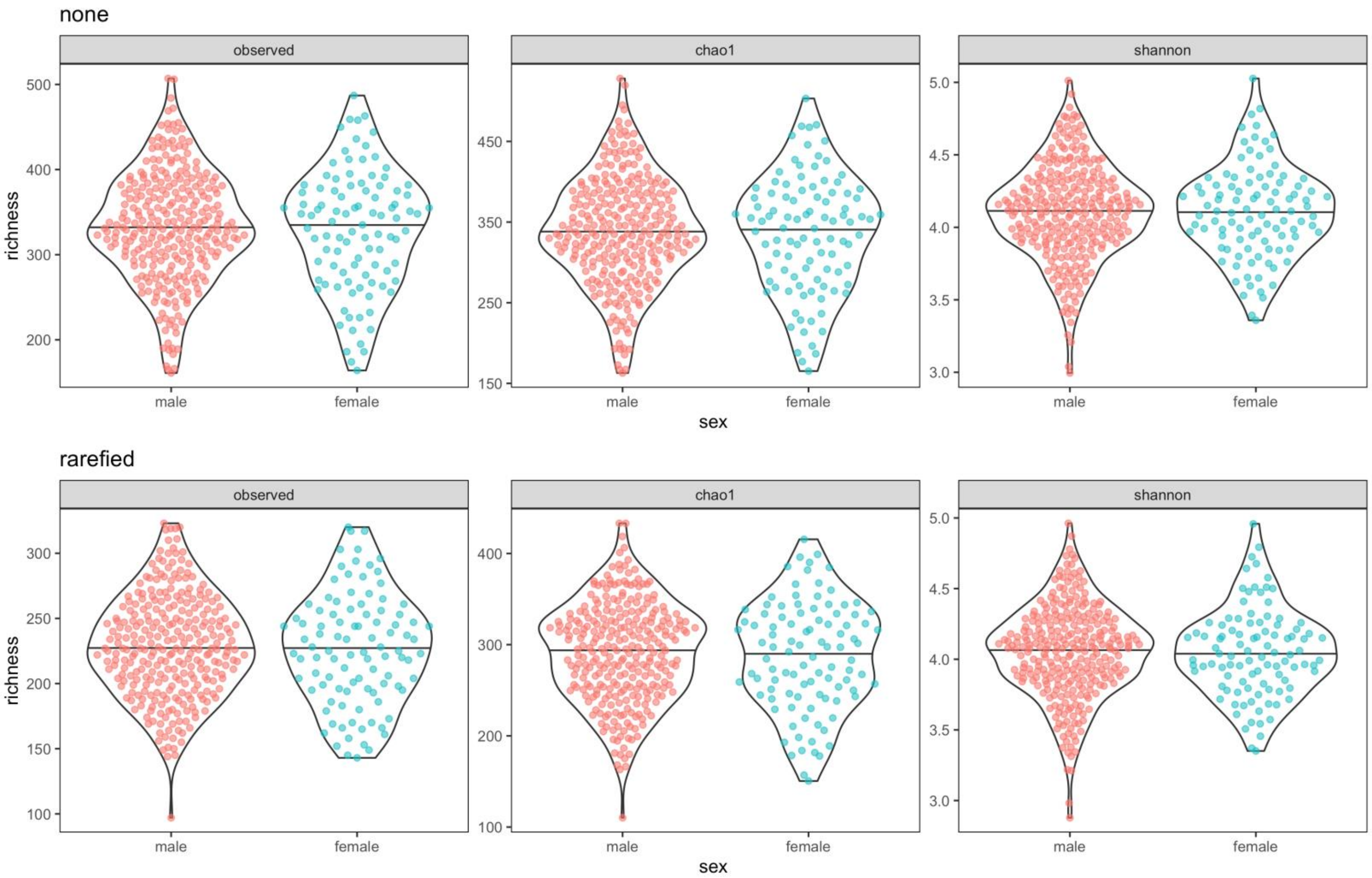

Supplement: Supplementary file 4 — Supplementary Material 4 [file 12903_2023_3448_MOESM4_ESM.pdf]
