# Supplementary material for: Oral microbiota analyses of paediatric Saudi population reveals signatures of dental caries
Source: BMC Oral Health. 2023 Nov 27;23:935. doi: 10.1186/s12903-023-03448-3 (PMC10683298; doi:10.1186/s12903-023-03448-3)
Supplement: Supplementary file 5 — Supplementary Material 5 [file 12903_2023_3448_MOESM5_ESM.pdf]

Supplementary Figure 4. Scatterplots showing the proportion of samples where a given OTU is observed at least once.

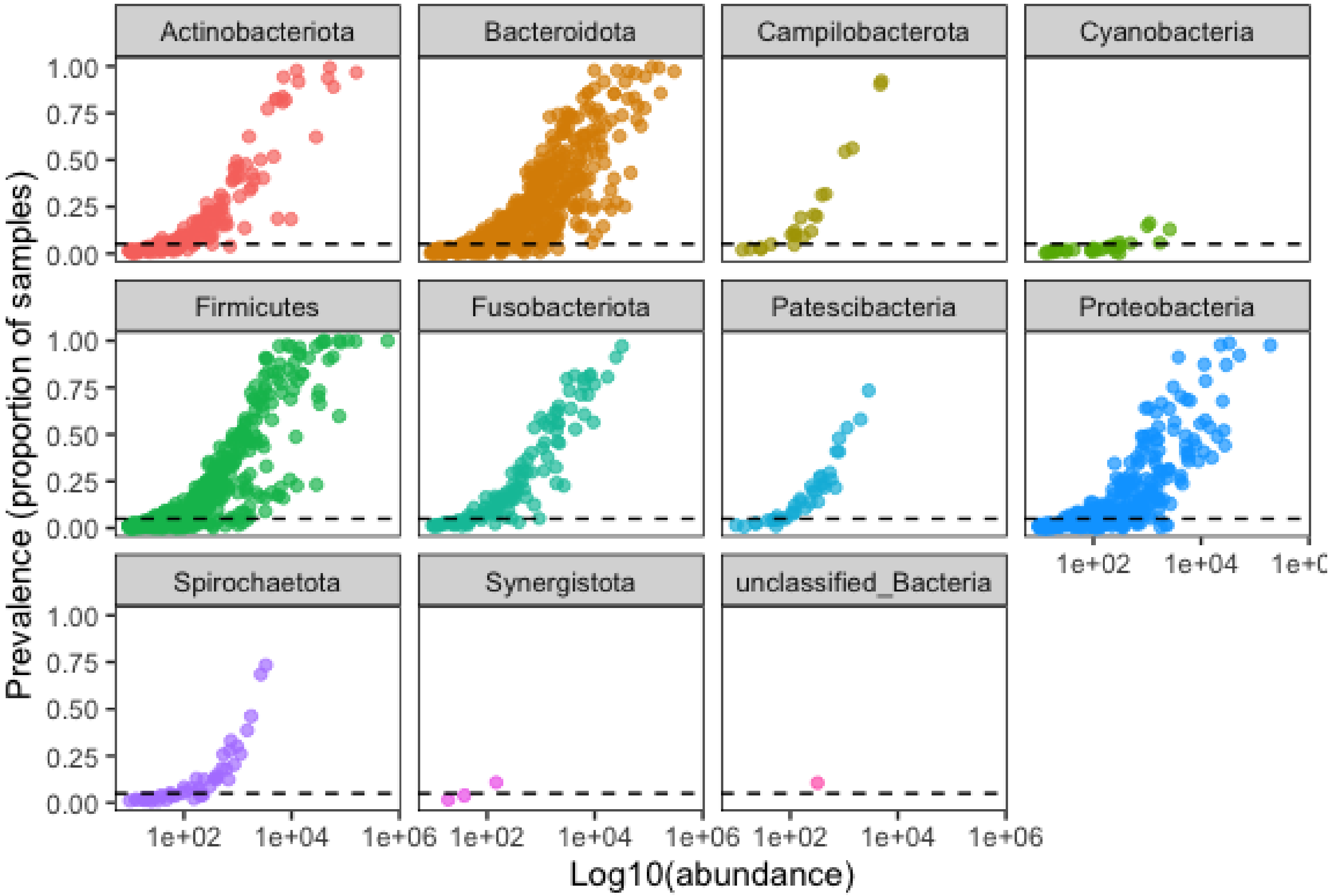

Each faceted plot shows the log10-transformed abundance on the x-axis and the prevalence on the y-axis for a given phylum. The dashed horizontal black line represents the prevalence filter at 0.05.
