# Supplementary material for: Oral microbiota analyses of paediatric Saudi population reveals signatures of dental caries
Source: BMC Oral Health. 2023 Nov 27;23:935. doi: 10.1186/s12903-023-03448-3 (PMC10683298; doi:10.1186/s12903-023-03448-3)
Supplement: Supplementary file 6 — Supplementary Material 6 [file 12903_2023_3448_MOESM6_ESM.pdf]

Supplementary Figure 3. Histogram distribution of reads per sample assigned to OTUs.

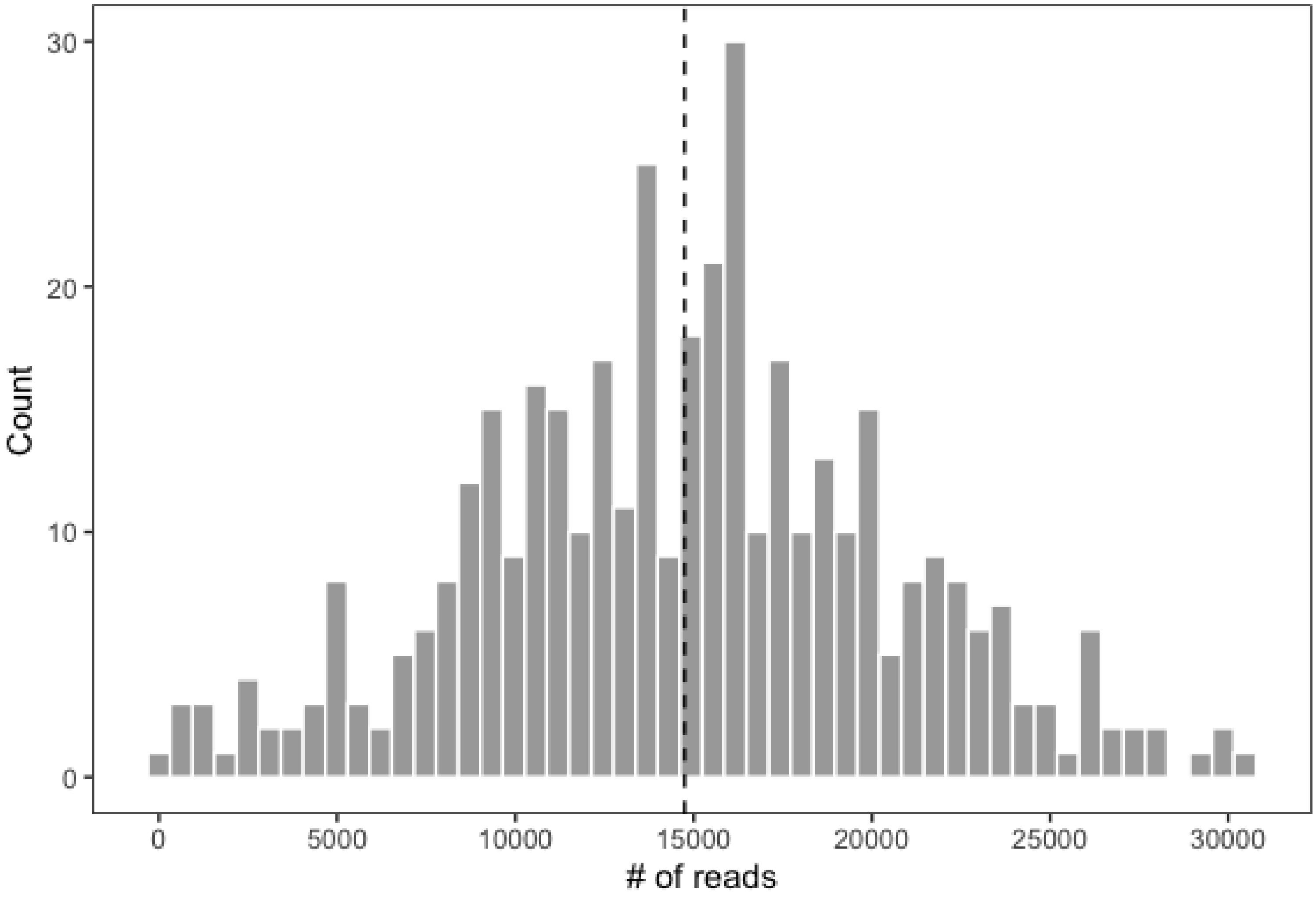

Total read number (x-axis), number of samples in each bin (y-axis) and mean read number per sample (dashed vertical black line).
