# Supplementary material for: Oral microbiota analyses of paediatric Saudi population reveals signatures of dental caries
Source: BMC Oral Health. 2023 Nov 27;23:935. doi: 10.1186/s12903-023-03448-3 (PMC10683298; doi:10.1186/s12903-023-03448-3)
Supplement: Supplementary file 7 — Supplementary Material 7 [file 12903_2023_3448_MOESM7_ESM.pdf]

Supplementary Figure 5. Rarefaction curves showing the number of observed OTUs as a function of sequencing depth.

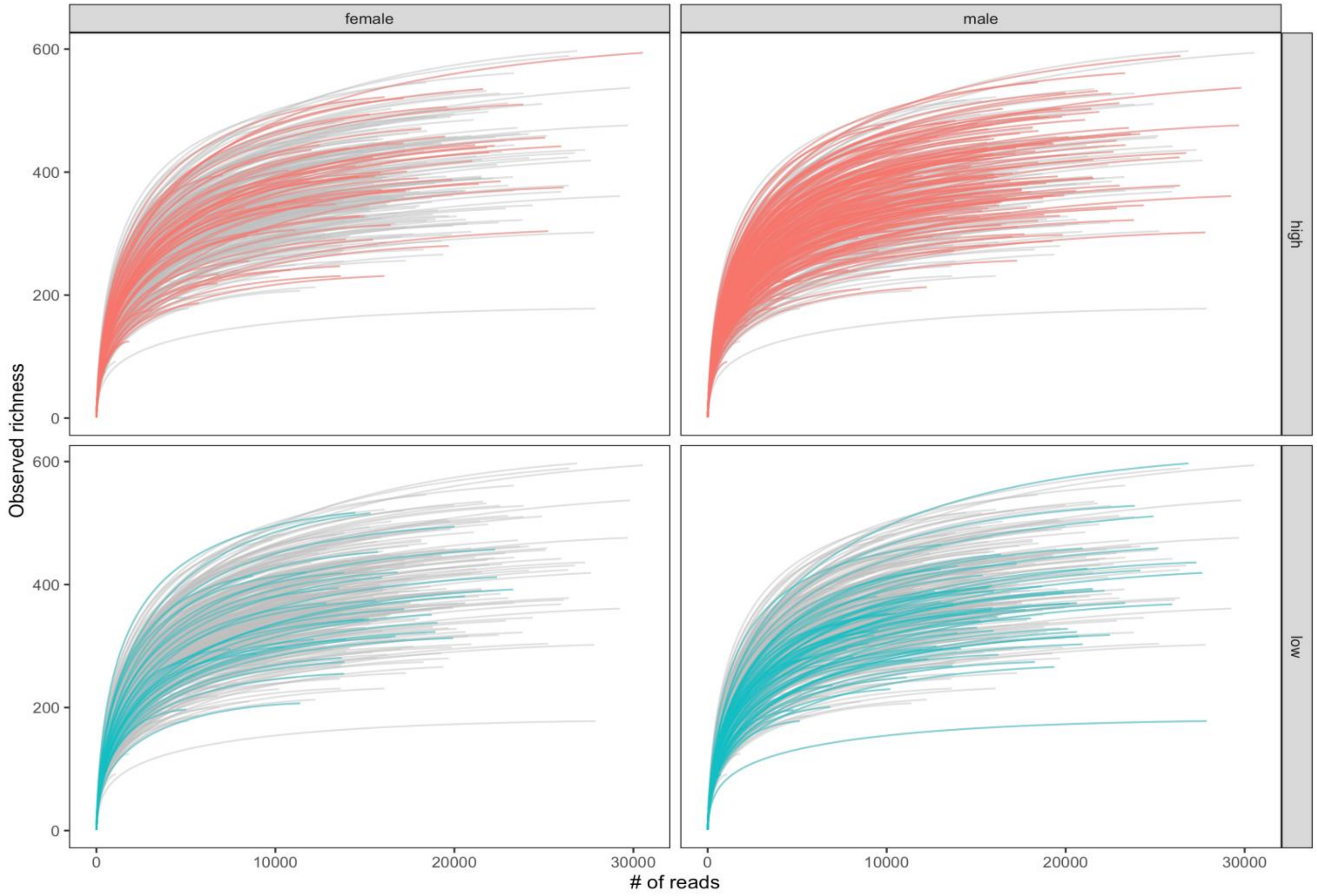

Vegan package was used to perform rarefaction with *ggplot2* and *gghighlight* packages used to create plots. Number of reads per subsample are shown on x-axis, and observed richness is shown on y-axis.
