# Supplementary material for: Oral microbiota analyses of paediatric Saudi population reveals signatures of dental caries
Source: BMC Oral Health. 2023 Nov 27;23:935. doi: 10.1186/s12903-023-03448-3 (PMC10683298; doi:10.1186/s12903-023-03448-3)

Supplementary Figure 7. Scatterplots showing all combinations of the five most significant principal components (PCs) identified using broken-stick test with percent of total variance explained by each PC presented on the respective axis. Each specimen is displayed as a dot, colored by **biological sex** (red, female; blue, male).

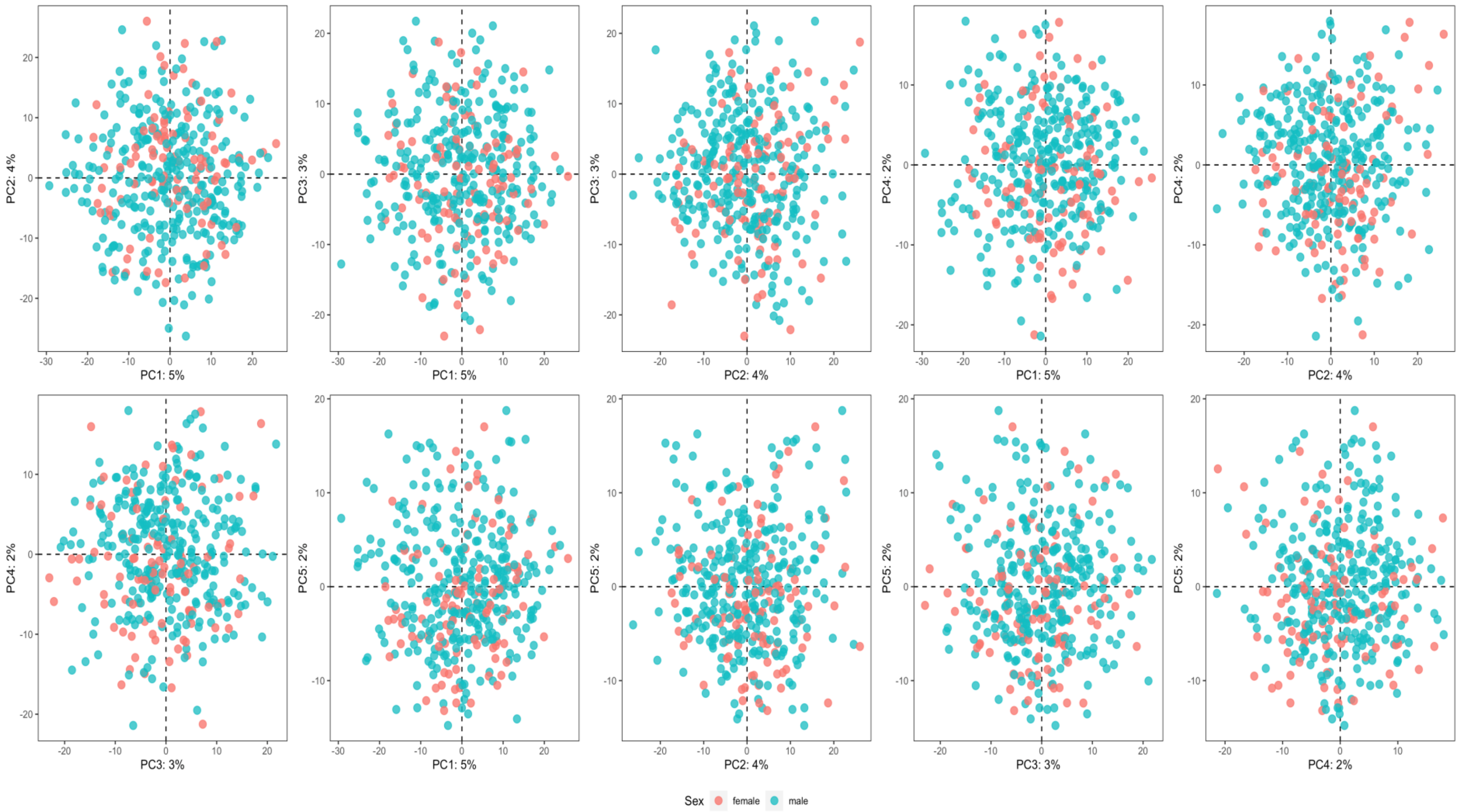

Supplement: Supplementary file 8 — Supplementary Material 8 [file 12903_2023_3448_MOESM8_ESM.pdf]
