# Supplementary material for: Oral microbiota analyses of paediatric Saudi population reveals signatures of dental caries
Source: BMC Oral Health. 2023 Nov 27;23:935. doi: 10.1186/s12903-023-03448-3 (PMC10683298; doi:10.1186/s12903-023-03448-3)

Supplementary Figure 10. Scatter and density plots of principal coordinates analysis scores using the Bray-Curtis dissimilarity, colored by sex. Each axis is displayed on the top & right axes with sample densities in PCoA space on each axis along the diagonal.

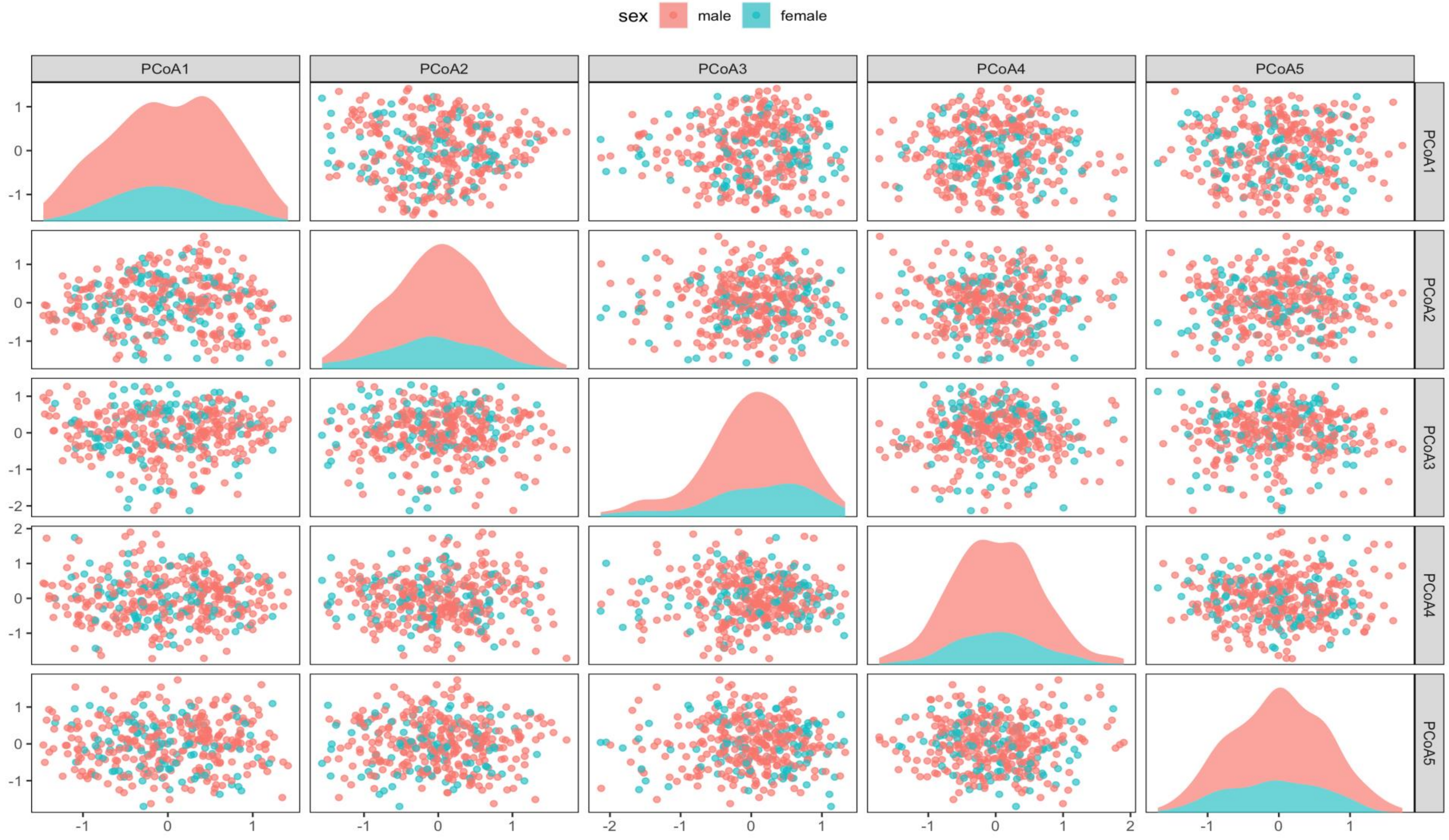

Supplement: Supplementary file 9 — Supplementary Material 9 [file 12903_2023_3448_MOESM9_ESM.pdf]
