# Supplementary material for: Oral microbiota analyses of paediatric Saudi population reveals signatures of dental caries
Source: BMC Oral Health. 2023 Nov 27;23:935. doi: 10.1186/s12903-023-03448-3 (PMC10683298; doi:10.1186/s12903-023-03448-3)

Supplementary Figure 8. PCA scatterplots showing all combinations of the five significant principal components (PCs) identified using the broken-stick test. The percent of the total variance explained by each PC is presented on the respective axis. Each specimen is displayed as a dot, colored by **Dental Caries status** (red, high; blue, low).

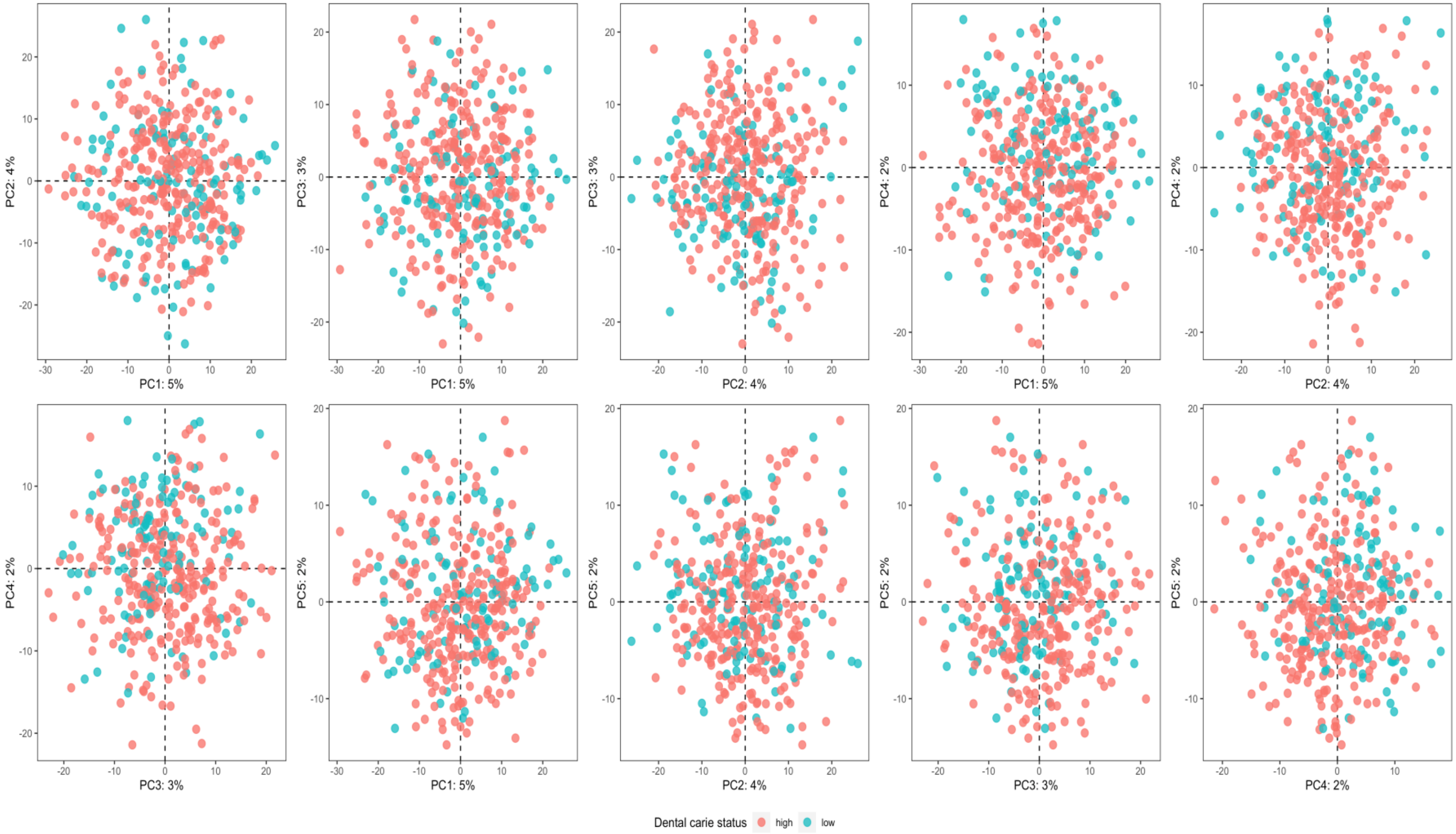

Supplement: Supplementary file 10 — Supplementary Material 10 [file 12903_2023_3448_MOESM10_ESM.pdf]
