# Supplementary material for: Oral microbiota analyses of paediatric Saudi population reveals signatures of dental caries
Source: BMC Oral Health. 2023 Nov 27;23:935. doi: 10.1186/s12903-023-03448-3 (PMC10683298; doi:10.1186/s12903-023-03448-3)

Supplementary Figure 11. Scatter and density plots of PCoA scores using the Bray-Curtis dissimilarity and colored by dental caries status. Each axis is displayed on the top and right axes with densities of samples in PCoA space on each axis along the diagonal.

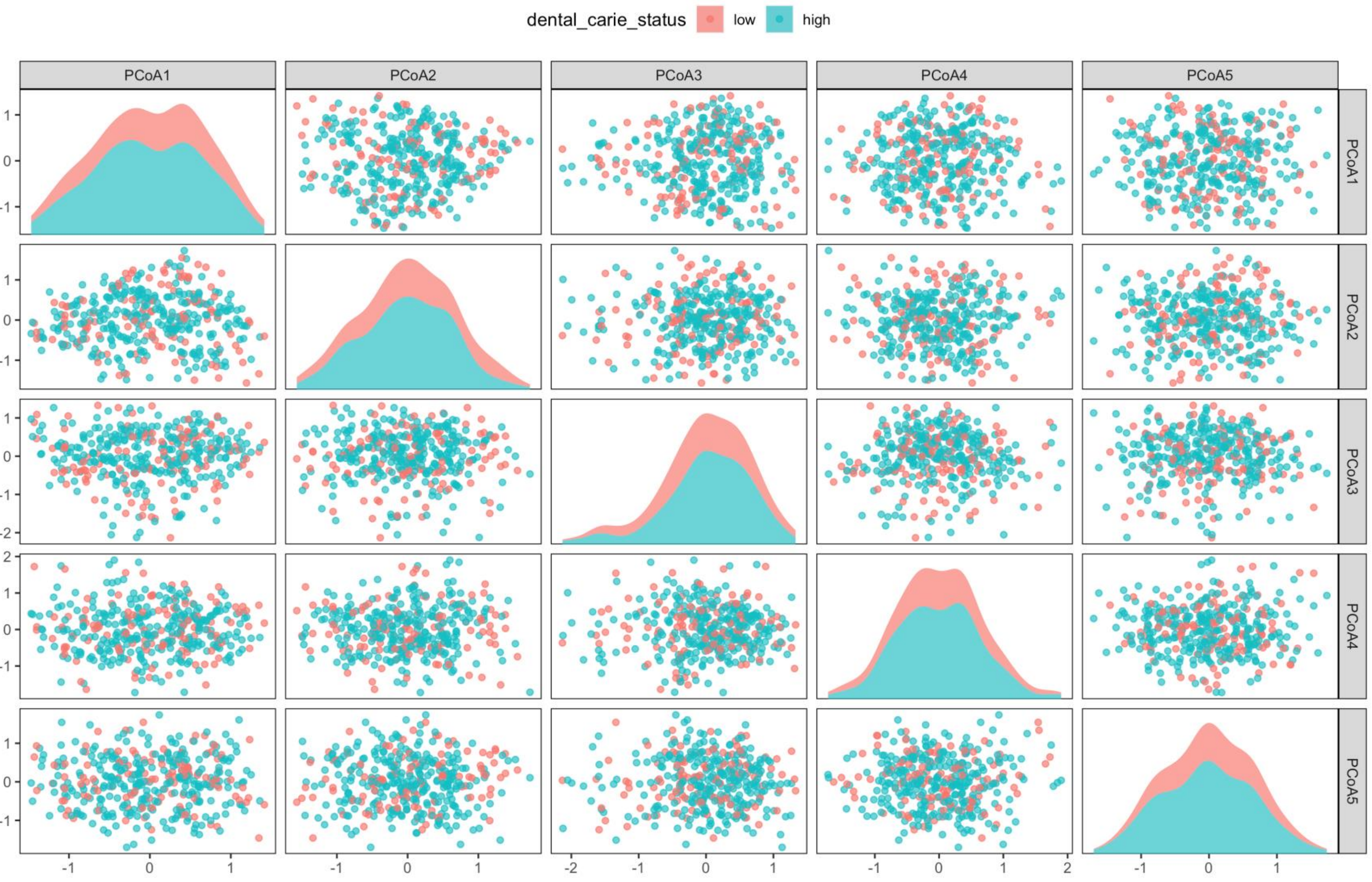

Supplement: Supplementary file 11 — Supplementary Material 11 [file 12903_2023_3448_MOESM11_ESM.pdf]
