# Supplementary material for: Oral microbiota analyses of paediatric Saudi population reveals signatures of dental caries
Source: BMC Oral Health. 2023 Nov 27;23:935. doi: 10.1186/s12903-023-03448-3 (PMC10683298; doi:10.1186/s12903-023-03448-3)
Supplement: Supplementary file 12 — Supplementary Material 12 [file 12903_2023_3448_MOESM12_ESM.pdf]

Supplementary Figure 14. Variable importance plot showing the top 10 features in the random forest model using all OTUs as features to predict Dental Caries status.

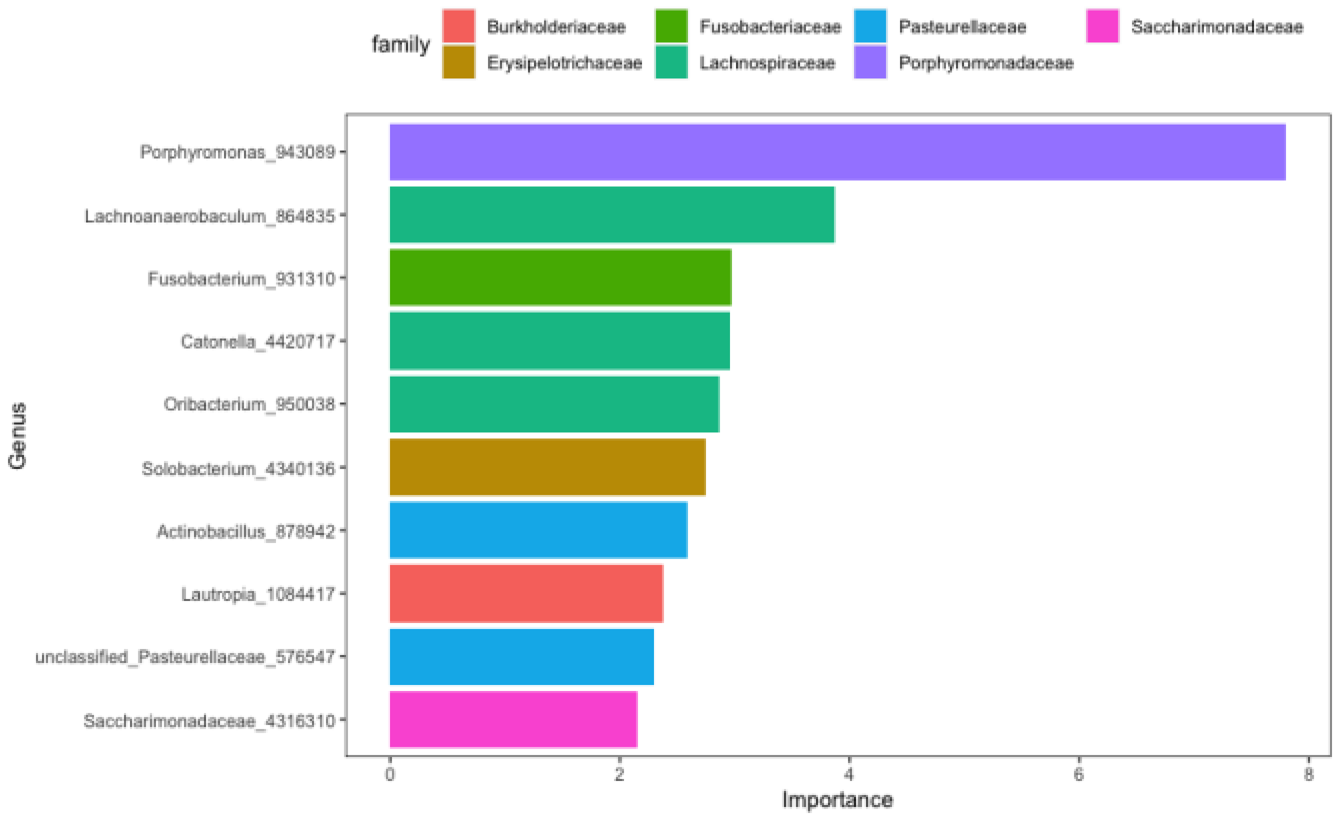

The importance score is presented on the x-axis, the genera are presented on the y-axis, and the bars represent families.
