# Supplementary material for: Oral microbiota analyses of paediatric Saudi population reveals signatures of dental caries
Source: BMC Oral Health. 2023 Nov 27;23:935. doi: 10.1186/s12903-023-03448-3 (PMC10683298; doi:10.1186/s12903-023-03448-3)

Supplementary Figure 12. Bar plots illustrate relative abundance of each phylum within each biological sex (female, top-left; male, top-right) and Dental Caries Status group (low, bottom-left; high, bottom-right). Each sample is presented on x-axis with relative abundance presented on the y-axis, and colors within each bar correspond to the observed phyla.

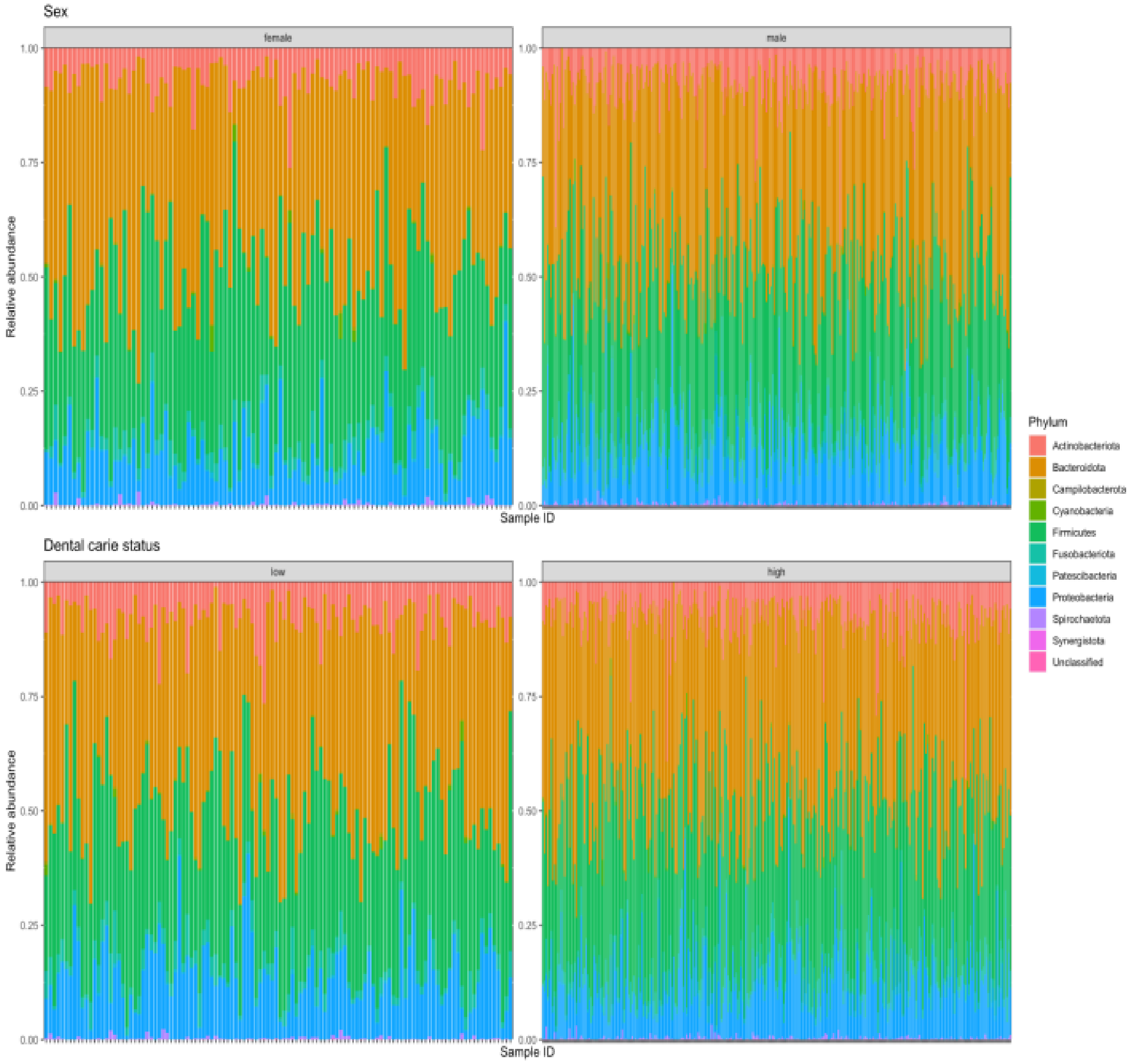

Supplement: Supplementary file 13 — Supplementary Material 13 [file 12903_2023_3448_MOESM13_ESM.pdf]
