# Supplementary material for: Oral microbiota analyses of paediatric Saudi population reveals signatures of dental caries
Source: BMC Oral Health. 2023 Nov 27;23:935. doi: 10.1186/s12903-023-03448-3 (PMC10683298; doi:10.1186/s12903-023-03448-3)
Supplement: Supplementary file 14 — Supplementary Material 14 [file 12903_2023_3448_MOESM14_ESM.pdf]

Supplementary Figure 13. Scatterplots showing mean AUC with different hyperparameter combinations in a random forest model with 5,000 trees using (a) differentially abundant OTUs (b) differentially all OTUs .

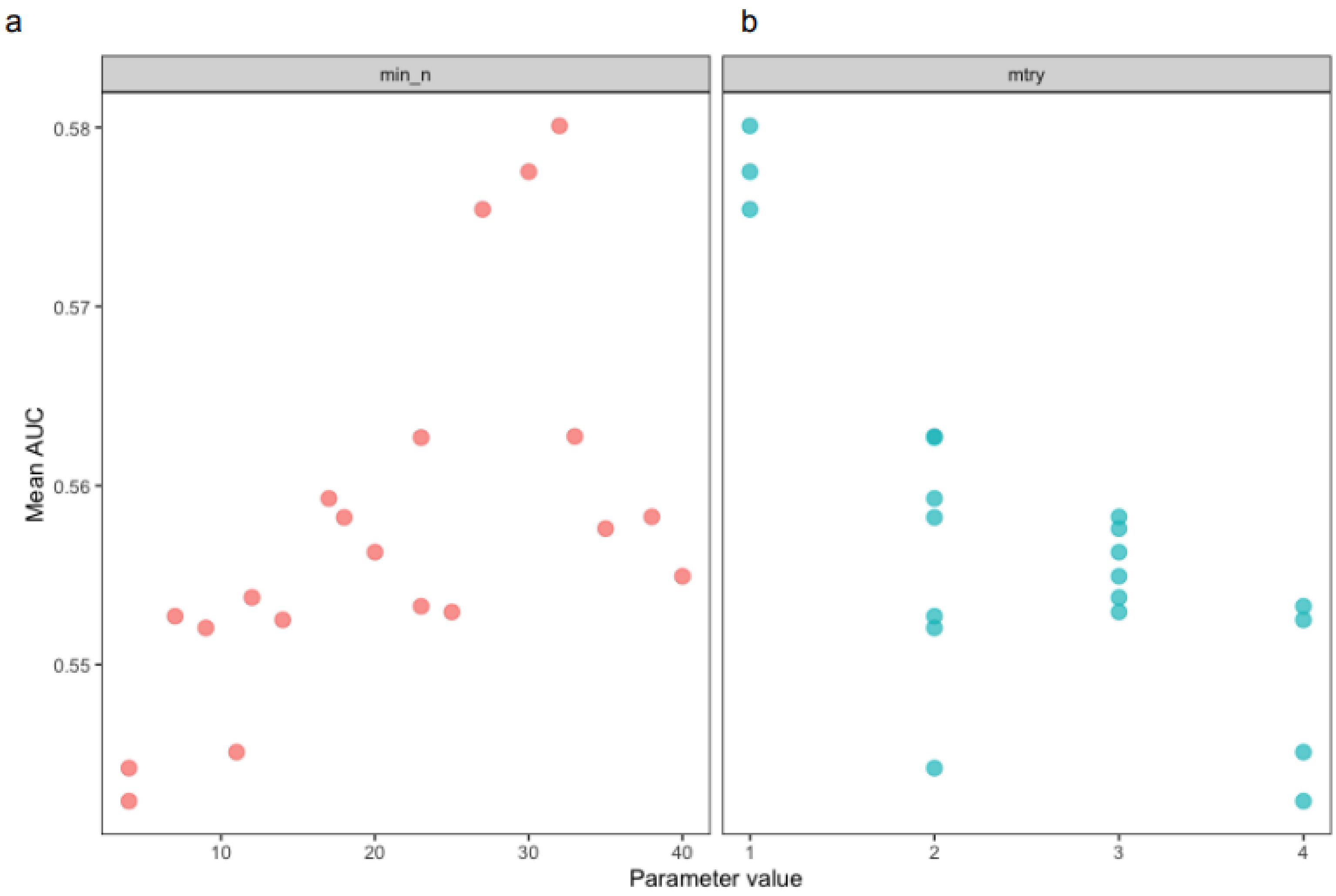

b

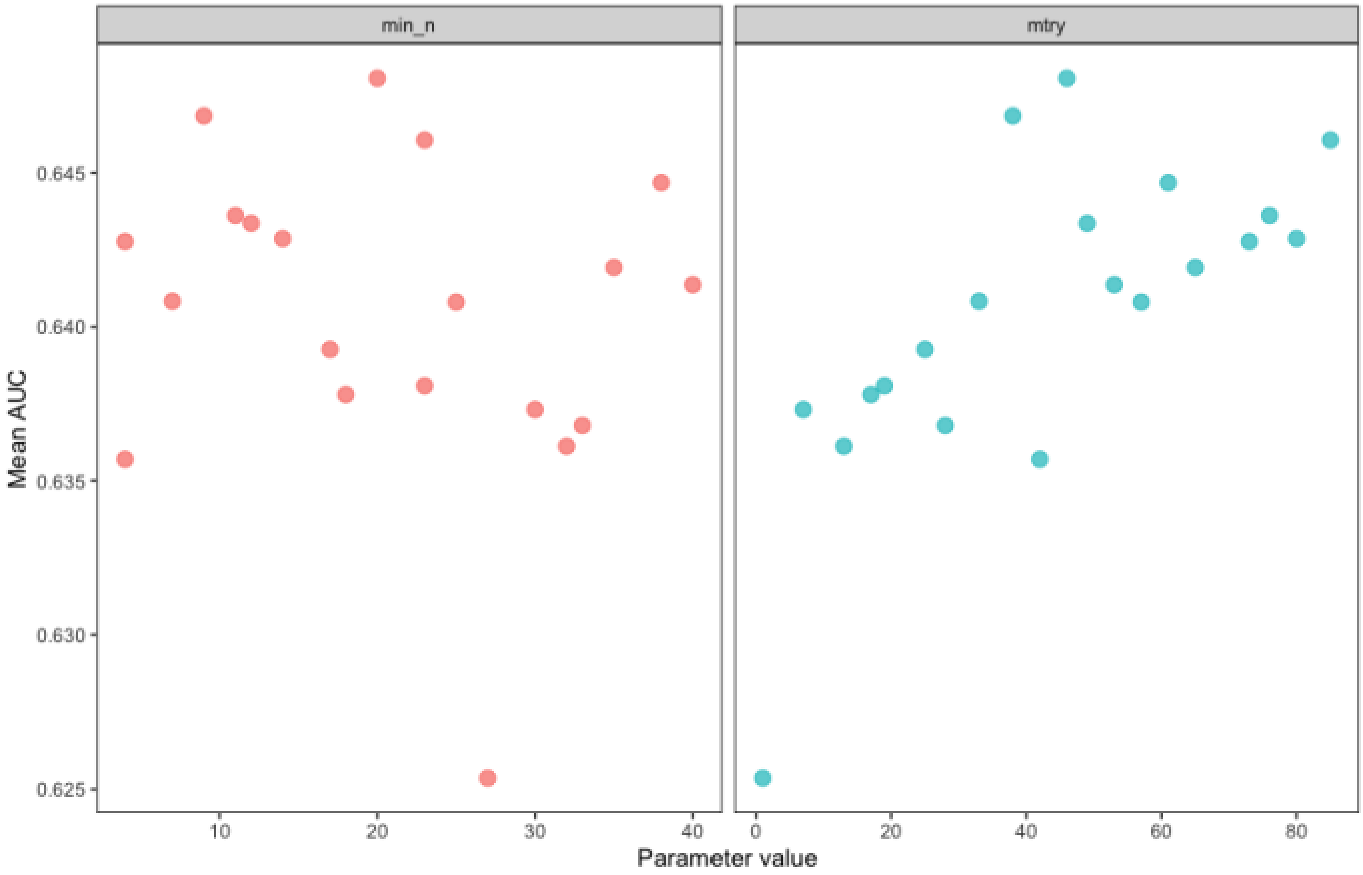
min_n
mtry
Mean AUC
0.645
0.640
0.635
0.630
0.625
10
20
30
40
0
20
40
60
80
Parameter value
