# Supplementary material for: Oral microbiota analyses of paediatric Saudi population reveals signatures of dental caries
Source: BMC Oral Health. 2023 Nov 27;23:935. doi: 10.1186/s12903-023-03448-3 (PMC10683298; doi:10.1186/s12903-023-03448-3)
Supplement: Supplementary file 15 — Supplementary Material 15 [file 12903_2023_3448_MOESM15_ESM.docx]

**Supplementary Table 1.** Differentially abundant OTUs between males and females (alpha = 0.1 level) identified using DESeq2. Taxonomic data for each identified OTU, estimated log2 fold change (LFC), and FDR adjusted p-values are shown

| Phylum | Class | Order | Family | Genus | LFC | P value adjusted |
| --- | --- | --- | --- | --- | --- | --- |
| Firmicutes | Bacilli | Lactobacillales | P5D1-392 | P5D1-392 | -0.252 | 0.051 |
| Proteobacteria | Gammaproteobacteria | Burkholderiales | Neisseriaceae | Kingella | -0.533 | 0.037 |
| Firmicutes | Clostridia | Lachnospirales | Lachnospiraceae | Stomatobaculum | 0.565 | 0.005 |
| Firmicutes | Bacilli | Lactobacillales | Carnobacteriaceae | Granulicatella | -0.245 | 0.037 |
| Proteobacteria | Gammaproteobacteria | Burkholderiales | Neisseriaceae | Unclassified Neisseriaceae | -0.856 | 0.008 |
| Proteobacteria | Gammaproteobacteria | Pseudomonadales | Moraxellaceae | Moraxella | 0.059 | 0.005 |
